# Recent developments in epigenetic cancer therapeutics: clinical advancement and emerging trends

**DOI:** 10.1186/s12929-021-00721-x

**Published:** 2021-04-12

**Authors:** Kunal Nepali, Jing-Ping Liou

**Affiliations:** 1 School of Pharmacy, College of Pharmacy, Taipei Medical University, 250 Wuxing Street, Taipei, 11031 Taiwan; 2 Biomedical Commercialization Center, Taipei Medical University, Taipei, 11031 Taiwan

**Keywords:** Epigenetics, Cancer, PROTACS, Multitargeting, Scaffolds, Inhibitors, Mechanisms, CRISPR/Cas9

## Abstract

Epigenetic drug discovery field has evidenced significant advancement in the recent times. A plethora of small molecule inhibitors have progressed to clinical stage investigations and are being explored exhaustively to ascertain conclusive benefits in diverse malignancies. Literature precedents indicates that substantial amount of efforts were directed towards the use of epigenetic tools in monotherapy as well as in combination regimens at the clinical level, however, the preclinical/preliminary explorations were inclined towards the identification of prudent approaches that can leverage the anticancer potential of small molecule epigenetic inhibitors as single agents only. This review article presents an update of FDA approved epigenetic drugs along with the epigenetic inhibitors undergoing clinical stage investigations in different cancer types. A detailed discussion of the pragmatic strategies that are expected to steer the progress of the epigenetic therapy through the implementation of emerging approaches such as PROTACS and CRISPR/Cas9 along with logical ways for scaffold fabrication to selectively approach the enzyme isoforms in pursuit of garnering amplified antitumor effects has been covered. In addition, the compilation also presents the rational strategies for the construction of multi-targeting scaffold assemblages employing previously identified pharmacophores as potential alternatives to the combination therapy.

## Background

Genome refers to the complete set of genetic information in the form of nucleotide sequence inside the DNA, whereas the epigenome refers to complex modifications inside the genomic DNA [1]. In simple terms, epigenetics involves a set of structural modifications within the nucleic acids and histone that do not involve a change in an individual’s genetic code [2–4] and can be termed as ‘on top’ or ‘in addition’ to genetics [5].

Epigenetic mechanisms regulate gene transcription and genomic stability and maintain normal cell growth, development, and differentiation [6–10]. As such, epigenetic regulation is a dynamic and reversible process and epigenetic modifications are carried out by writers (DNMTs, HATs, ubiquitin E3 ligases and HMTs) that catalyze the addition of epigenetic marks onto either DNA or histone tails, readers (bromodomains) that recognizes or are recruited to a specific epigenetic mark and erasers (HDACs, KDMs and deubiquitinating enzymes) that removes the epigenetic marks [11–21].

Though epigenetics is a key component of an organism’s normal development, from embryonic development through adulthood, epigenetic dysregulation can significantly contribute to the origin and progression of human diseases such as cancer, cardiovascular diseases, metabolic diseases and neurological diseases. Extensive explorations conducted to enhance the understanding of the epigenome reveals that localised differences existing in epigenetic states of normal and disease tissues can be utilized as disease biomarkers [22–26].

Literature precedents indicate that all the three families of epigenetic proteins—readers, writers, and erasers are druggable targets. This disclosure coupled with the improved understanding of epigenetics in diverse complications dramatically spurred and expedited the translational investigation of the epigenetic inhibitors. In particular, exhaustive investigations predominantly on small-molecule inhibitors were carried out at the clinical level and the subsequent efforts have culminated in the identification of efficacious inhibitors, with some of them being used in the clinic currently. To add on, the preclinical and preliminary studies have also comprehensively explored the epigenetic tools (DNMT/HDAC/LSD1/DOT1L/BET/EZH2 inhibitors) in pursuit of leveraging enhanced antiproliferative effects. Albeit the clinical stage investigations have been appropriately directed towards the evaluation of the epigenetic inhibitors as single agents, a significant proportion of the efforts is also covered by the studies inclined towards the utilization of the epigenetic tools as a part of combination regimens. This information raises a critical question regarding the therapeutic credibility of some of the epigenetic inhibitors as single agents to attain conclusive benefits in cancer. The doubts are further strengthened by the fact that only seven drugs have approved till date despite the epigenetic targets being at the forefront of the strategized explorations. Nevertheless, the medicinal chemist at the preclinical/preliminary level has been quite proficient to employ rational drug design approaches to maximize the benefits of the predefined pharmacophore models of the epigenetic targets. Indeed, the preclinical/preliminary findings (section) bears a relatively higher degree of fascination for the researchers as efforts invested have not just been confined to elucidate the mechanistic insights responsible for exerting antitumor effects via inhibition of the epigenetic targets, rather the chemist has looked beyond this strategy to attain favourable effects via degradation of the proteins also (PROTACS). Sagaciously evidenced on the literature precedential basis, degradation of the target proteins can be achieved at low exposures by PROTACs (protein degraders) owing to their catalytic mode of action and this emerging approach is likely to steer the wheels of the drug discovery field towards the class of degraders bearing appropriately installed epigenetic tools in the near future. For the selective targeting, the concept of antibody–drug conjugates have also attracted the eyeballs of the researchers working in the field of epigenetic inhibitors. This strategy of targeted drug delivery is anticipated to overcome the issue of systemic toxicity and narrow therapeutic window that limits the clinical use of the available epigenetic inhibitors. CRISPR/Cas9-based strategies to target the cancerous epigenetic regulators also represent an emerging potential approach that is being foreseen as a tool to correct genetic mutations. Other than this, the approach of multitarget assemblage construction has continued garnering significant attention to extract enhanced antitumor effects via concomitant inhibition of the biochemically correlated targets and is also conceived to be one of the preferred futuristic strategies as a potential alternative to the combination therapy. To sum up, it is highly likely that the ship of epigenetic inhibitors will sail through the implementation of the aforementioned approaches.

Despite the significant promise demonstrated by the aforementioned strategies, there is no denying the fact that the conventional approaches will continue receiving tantamount attention of the research groups for the development of new inhibitors. Fragment stitching approach on existing drugs coupled with lead modification studies ascertaining the impact of scaffold installation, regiovariation, bioisosteric replacement, structure simplification approach, structure rigidification approach and other subtle structural variations on the activity profile exemplifies some of these potential approaches.

In light of the current scenario and the amount of efforts currently being invested in this field, it is highly likely that this decade might evidence the therapeutic growth of a handful of epigenetic drugs presently undergoing efficacy and safety evaluations at the clinical level and many new agents might enter the clinic. This review article presents an update of FDA approved epigenetic drugs along with the epigenetic inhibitors undergoing clinical stage investigations. The compilation also encompasses a detailed discussion of the rational strategies that can prove to be instrumental in the development of new inhibitors. The covered literature in this review indicates that the future attempts in the epigenetic drug discovery filed needs to headed in the following directions: (i) explorations of natural product based libraries for the development of non-nucleoside based DNMT inhibitors (ii) initiation of parallel programs on non-metal chelating type HDAC inhibitors as well as anilides to transpose the focus from hydroxamic acid type scaffolds owing to the pharmacological liabilities associated with latter class (iii) exhaustive studies needs to be conducted to ascertain the expression level of epigenetic enzymes in diverse malignancies (iv) fabrication of selective isoform inhibitors of HDAC to extract amplified anticancer effects despite of the fact that the clinical success, till date, have only been attained through pan HDAC inhibitors (v) exploration of additional structural templates other than the framework of tranylcypromine to expand the size of LSD1 inhibitors pipeline (vi) design of dual EZH1/EZH2 inhibitors in view of the fact EZH1, complements EZH2 in mediating H3K27 methylation and is also endowed with HMT activity. (vii) Expanding the size of the libraries of DOT1L inhibitors (viii) utilization of the existing chemical architectures of BET and HDAC inhibitors in the PROTAC model and antibody–drug conjugate model (ix) explorations of combination of epigenetic inhibitors with immunotherapy.

## Epigenetics and cancer

Epigenetic processes comprises of inherited, somatic and reversible changes in gene expression in cancer cells. DNA methylation, histone modification (acetylation, methylation, phosphorylation, etc*.*) and noncoding RNAs are the major epigenetic mechanisms that control gene activity leading to a number of complex cancers [4]. In most of the cancers, DNA is hypomethylated along with the hypermethylation at other sites [27]. The two anomalous processes i.e. hypomethylation and hypermethylation activates oncogenes and inhibits the tumor suppressor genes, respectively [28]. Apart from methylation process, histone modification is another process that plays important role in cancer. Histone modifications control the active and inactive state of chromatin which ultimately influences the gene expression within the former region [29]. MicroRNAs are responsible for degradation of mRNA as well as inhibition of target mRNA through respective complementary base pairing and partial base pairing [30]. All these epigenetic changes start taking place a long time ago before the occurrence of cancer and are considered accountable for any genetic changes in cancer, also labelling them as “first hits” for tumorigeneses [27].

### Role of DNA methylation in cancer cells

DNA methylation is an epigenetic process that can be described as the covalent transfer of methyl groups to the fifth carbon of cytosine (5-mC) within 5′-CpG-3′ dinucleotides catalysed by DNMTs with SAM as the methyl donor [31, 32]. In mammals, three major types of DNMT enzymes are found, DNMT1, DNMT3a, and DNMT3b. DNA methylation is appointed as an epigenetic marker that manage the time and location of genes expression in both normal and diseased cells [33]. In cancers like breast, colon, esophageal, lung, pancreas, ovary, prostate, and other cancers, altered patterns of DNA methylation have been observed [34]. The hypomethylation results in re-expression of silenced genes and genomic instability leading to demethylation of two elements that consists of long interspread transposable elements and short interspread transposable elements [35, 36]. Besides hypomethylation, the outcome of hypermethylation is the silencing of TSGs, such as P15INK4b, P16INK4a, P14ARF, CDH1 or EXT1 [37]

### Acetylation and deacetylation

It is well known that the acetylation and deacetylation of N-terminal of lysine residue of histone is a critical part of gene regulation and the process is controlled by two enzymes HAT or HDAC [38]. The acetylation results in condensed chromatin structure leading to cell transcription promotion while deacetylation leads to relaxed chromatin causing suppression of gene transcription [39]. This balance between HAT and HDAC manages the chromatin structure and gene expression [40]. Any imbalance in the activity of HAT and HDAC results in cancer. HAT enzyme is associated with various transcription factors like GCN5-related Nacetyltransferase, MYST, and cAMP response element binding protein (CREB/p300) families. Dysbalances in histone acetylation has been evidenced in Rubinstein–Taybi syndrome, glioblastomas, lung cancers, and AML [41]. On the other side any alteration in expression of different isoforms of HDACs also causes various cancers like increased levels of HDAC 2 and 3 is observed in colon cancer, rise in levels of HDAC 1 is observed in gastric cancer while in lung cancer reduced expression of HDAC5 and HDAC10 is observed [42]. Furthermore, over expression of HDAC 1 is reported in prostate and esophageal squamous cell carcinoma [43].

### Histone methylation and demethylation

The extent and location of methylation and demethylation of histones is another important parameter that controls the gene transcription. Both lysine and arginine residues are prone to methylation but lysine residues H3 and H4 of histone tail are more liable to methylation [44]. The known sites for methylation that controls gene activation are H3K4, H3K48 and H3K79 whilst H3K9 and H3K27 are the sites for gene inactivation [45]. A group of proteins containing the SET (enhancer of-zeste, trithorax) called HMT is required by lysine for methylation process [46]. Histone demethylation enzymes known as KDMs are divided into two groups based on their sequence homology and catalytic mechanism. These includes FAD-dependent amine oxidases superfamily called LSDs [47] and (2) the JmjC domain, contains α-ketoglutarate-dependent enzymes, KDMs and Fe(II) [48]. Any irregulatory in epigenetic effects of methyltransferase enzymes can result in a variety of malignancies [49].

### Protein phosphorylation

Phosphorylation takes place at side chains of serine, threonine, and tyrosine via phosphate ester linkages in which histidine, lysine and arginine squeeze through the phosphoramidate linkages, and through the mixed anhydride linkages that occur at amino acids, aspartame acid and glutamate [50]. Phosphorylation helps in regulation of a number of biological processes like various signalling pathways, gene expression, cell division, etc*.* while majority of the cellular functions that includes energy storage, morphological changes, protein synthesis, gene expression, signaling factor release, muscle contraction, and biochemical metabolism are controlled and managed by phosphorylation [51]. A number of signalling pathways are controlled by protein and lipid kinases for regulation of normal cell functions [52–56]. The abnormalities in activity of kinases results in a variety of pathological events, amongst which cancer is the most prominent [52, 53, 56].

### Ubiquitination

Ubiquitin system in body consists of three main enzymes ubiquitin-activating enzymes (E1s), binding enzymes (E2s), ligases (E3s), and degrading enzymes [57]. Ubiquitination performs the following functions localization, metabolism, function, regulation and degradation of proteins. The diminished activity of E3 ubiquitin ligase due to some mutations can cause various cancers like renal cell carcinoma, breast cancer, etc*.* On the other hand, the increase in ubiquitination activity results in cervical cancer. Further total elimination of ubiquitination will lead to colorectal cancer and glioblastoma [58].

### SUMOylation

Small ubiquitin-like modified proteins (SUMO) are very similar to ubiquitin proteins as the name signifies. The process of SUMOylation of target proteins results in varied localization and binding partners which ultimately influences the three main parameters: the stability of protein, its transport between cytoplasm and nucleus and regulation of transcription [59]. The promyelocytic leukaemia protein and the oncogenic fusion protein PML–retinoic acid receptor-α are first discovered substrates of SUMO and the occurrence of cancer due to SUMO can be well explained on the basis of the above-mentioned substrates. An infrequent haematological malignancy occurs due to PML-RARα that is called acute promyelocytic leukaemia. The SUMOylation of PML, when distorted, leads to the expression of PML-RARα thus causing APL. SUMOylation is neither tumour promoting nor tumour suppressive rather it is a required process for all cells [60].

### Noncoding RNAs in cancer cells

Noncoding RNA is a novel class of genes that control regulatory functions in normal development of cells which get changed in tumor cells. Small nucleolar RNA, PIWI-interacting RNA, small interfering RNA, and microRNA are some of the examples of noncoding RNAs and exhibits functions like transcriptional and posttranscriptional gene silencing via selective base pairing with their targets [4]. Approximately 60% of genes that codes for different proteins and maintains the cellular processes are regulated by miRNAs [61]. Recently, it is reported that miRNAs behave as oncogenes by altering the tumor suppressing proteins or TSGs by modulating the levels proteins that exhibit oncogenic potential [62]. Although, all kinds of ncRNAs exhibits important functions in maintenance of different cellular processes but any irregularity in their function and expression may lead to carcinogenesis [63]. Another ncRNA is small nucleolar RNAs whose dysregulation is reported to be involved in tumorigenesis [64]. For instance, snoRNA42 (H/ACA snoRNA) is a type of snoRNA which is overexpressed in lung cancer [65].

## Epigenetics tools for cancer therapy in cancer

**Fig. 1:**
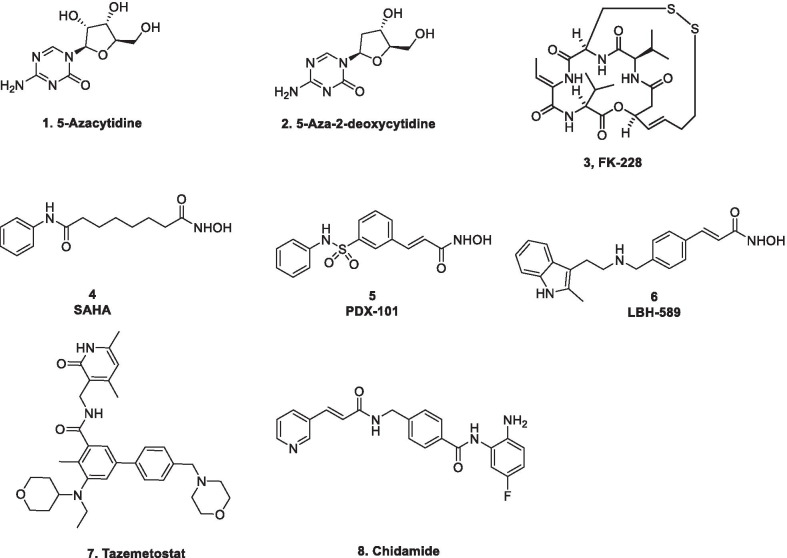
FDA and CFDA approved inhibitors of the epigenetic targets

Owing to the well-established role of epigenetic dysregulation towards the origin and progression of cancer, lot of efforts have been invested towards the development of epigenetic drugs for the treatment of cancer. The extensive research conducted on small molecule inhibitors as epigenetic tools (DNMT inhibitors, HDAC inhibitors, DOT1L inhibitors, LSD inhibitors, EZH2 inhibitors, BET inhibitors) makes it evident that the epigenetic proteins are druggable targets. At present, seven agents in three epigenetic target classes (DNMT, HDAC and EZH2 inhibitors) have been approved by the US FDA for the treatment of diverse malignancies (Fig. 1) and a wide range of epigenetic-based drugs are undergoing clinical trials. These include 5-azacytidine (**1**, DNMT inhibitor approved for the treatment of MDS) [66], 5-Aza-2-deoxycytidine (**2**, DNMT inhibitor approved for the treatment of MDS) [66], FK-228 (**3**, HDAC inhibitor approved for the treatment of refractory CTCL) [67], SAHA (**4**, HDAC inhibitor approved for the treatment of refractory CTCL) [68], PXD101 (**5**, HDAC inhibitor approved for the treatment of refractory PTCL) [69], LBH589 (**6**, HDAC inhibitor for the treatment of multiple myeloma) [70] and tazemetostat (**7**, EZH2 inhibitor approved for the treatment of metastatic or locally advanced epithelioid sarcoma) [71]. Other than the aforementioned FDA approved agents, an anilide type HDAC inhibitor, chidamide (**8**), has also been approved by CFDA to treat patients with R/R PTCL [72].

### DNMT inhibitors

**Fig. 2:**
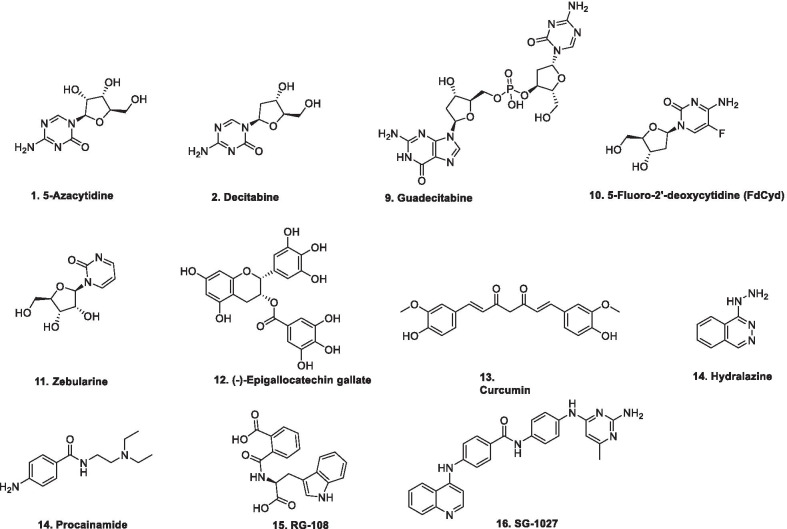
DNA methyl transferase inhibitors (nucleoside and non-nucleoside based)

DNMT blockade is considered to be a successful strategy for the prevention of aberrant DNA hypermethylation. DNMT inhibitors reactivate the aberrantly methylated TSG, thereby causing cancer cells reprogramming that ultimately lead to proliferation arrest and cell death [73, 74]. Literature precedents indicate that various compounds have been identified both at the preclinical as well as clinical level that can erase abnormal methylation patterns via irreversible inhibition of DNMTs, causing proteosomal degradation [75, 76]. This degradation then leads to attenuation of the neoplastic cell phenotype by inducing cell differentiation and tumor cell death [73, 74, 77]. Generally, the inhibitors of DNMT are categorized in to two classes: nucleoside analogs and non-nucleoside analogs (Fig. 2).

#### Nucleoside analogs

Comprising of a modified cytosine ring (nitrogen in place of a carbon at 5), nucleoside analogs can be converted to nucleotides and get incorporated into newly synthesized DNA or RNA. The enzyme DNMT gets bound with the analogs through the formation of covalent complexes that leads to the DNA methylation inhibition [78]. 5-Aza cytidine and decitabine (5-aza-2′-deoxycytidine) represents the nucleoside analogues that have been approved by FDA for the treatment of AML and MDS [74, 77]

#### 5-Aza cytidine

5-Aza cytidine (Cytidine analog) is a ribonucleoside analog that undergoes phosphorylation to get incorporated in to the RNA. 5-Azacytidine can also get incorporated into DNA via the ribonucleotide reductase pathway. At present, 5-Aza-cytidine is undergoing several clinical stage investigations for diverse malignancies. A phase 3 clinical trial of azacitidine conducted in patients with higher-risk MDS demonstrated that azacitidine (75 mg/m^2^ per day, 7 days every 28 days) increased the OS in comparison to conventional care [79]. The phase 4 clinical investigation of azacytidine was also conducted in patients with higher-risk MDS. The study design involved the administration of azacitidine 75 mg/m^2^/day for 7 days/28-day cycle for up to six cycles. The results of the study demonstrated that out of the 44 patients enrolled for the study, response-evaluable patient (n = 33) did not achieve complete remission or partial remission. However, haematological improvement was attained in 50% patients. RBC transfusion independence was attained in 12 of 32 patients and platelet transfusion independence was achieved in 7 of 18 patients. Neutropenia (52%) and leukopenia (39%) was observed as the common grade 3–4 TAAEs [80] (NCT01201811). In a phase 3 study assessing the benefits of azacitidine over the conventional care regimens in old patients with newly diagnosed AML, it was observed that the treatment with 5-azacytidine (N = 129, 8.9 months) led to remarkable prolongment of the median OS versus conventional care regimens (CCR) (n = 133, 4.9 months) [81] (NCT01074047)**.**

Azacytidine has also been evaluated in various combination regimens**.** The combination of azacytidine (75 mg/m^2^) and standard induction therapy was found to be feasible in older patients with AML [82] (Phase 2, NCT00915252)**.** The phase 2 trial of 5-azacytidine with lirilumab (BMS-986015) in patients with refractory/relapsed AML was terminated as the response rate did not meet the anticipated minimum 30%. (NCT02399917). In a phase 2 trial evaluating the combination of 5-azacytidine and sorafenib in older patients (n = 27) with untreated FLT3-ITD Mutated AML, 78% ORR, 26% CR, 44% Cri/CRp and 7% PR was observed. The median OS was 8.3 months and 9.2 months in the 19 responders. Overall, the results demonstrated that the combination was well tolerated in the specified population [83] (NCT02196857). The study evaluating the advantages of sequential azacitidine and lenalidomide in subjects with R/R AML demonstrated that this regimen was only effective in a minority of patients (only 11%). Moreover, significant toxicity was evidenced in some of the cases and three treatment-related deaths occurred [84] (NCT01743859). In an investigation evaluating the efficacy of sequential azacitidine and lenalidomide, or azacitidine in old patients with newly diagnosed AML, it was deduced that the regimen (sequential azacitidine and lenalidomide) is not favoured over azacitidine administered in conventional dose and schedule. With sequential azacitidine and lenalidomide, one-year survival was 44% (95% CI: 28, 60%) where as the one year survival with azacitidine only was 52% (95% CI: 35, 70%) [85] (NCT01358734). In a phase II study conducted in elderly population of higher risk MDS or AML that were, as such, considered unfit for intensive chemotherapy, the combination of azacytidine with escalated doses of lenalidomoide was not well tolerated and was discontinued in majority of the patients owing to toxicity issues. However, some positive results were observed in terms of cytogenetic response in the study. [86] (NCT01088373). In a phase 2 study conducted recently, prophylactic low-dose azacytidine and donor lymphocyte infusions following allogeneic hematopoietic stem cell transplantation for high-risk AML (n = 30 patients) and MDS (n = 10 patients) was evaluated. The study results demonstrated that azacytidine was well tolerated but was discontinued in 20 patients owing to graft-versus-host disease and relapse. The overall and disease-free survivals were 65.5% (CI 95% = 48.2–82.8) at 2 years. On the basis of these results, it was concluded that 5-azacytidine demonstrated potential as a prophylactic treatment to reduce the risk of post-transplantation relapse [87] (NCT01541280). A clinical study for assessing the efficacy of the combination of lirilumab and azacitidine in patients with MDS was conducted and 10 patients were enrolled for the investigation. Two patients achieved CR, 5 achieved marrow CR and 3 demonstrated SD. Grade > 3 AEs (infection or neutropenic fever) were observed in five patients. Overall, the combination of azacitidine and lirilumab demonstrated clinical activity [88] (NCT02599649). Azacitidine in combination with midostaurin in subjects (n = 14 in phase 1 and n = 40 in phase 2, enrolled) with AML and high risk MDS was also evaluated. The study design involved the administration of azacytidine 75 mg/m^2^ on days 1–7 and midostaurin 25 mg bid (in cohort 1 of phase I) or 50 mg bid (in cohort 2 of Phase I and in Phase II) orally. The results of the study demonstrated that the combination is safe as well as effective for patients with FLT3 mutations that were not previously treated with other FLT3 inhibitors [89] (NCT01202877). In a phase 2 evaluation assessing the combination of azacitidine and etanercept for the treatment of MDS, azacitidine (75 mg/m^2^/day for 7 days) was administered to twenty-three patients in combination with etanercept (25 mg sc twice a week for 2 weeks every 28 days). The results of the study indicated that a total of 14 patients responded, with CR evidenced in five patients and PR in 8 patients. A hematologic improvement of neutrophils was observed in 1 patient. Overall, the combination was deduced to be endowed with favourable trends in comparison to azacytidine alone. [90]

In a phase 1 study of azacytidine (monotherapy, combination with carboplatin or nab-paclitaxel) conducted in patients with R/R solid tumors, RP2D was determined as 300 mg (every day, days 1–14/21). PR (three/eight) and SD (four/eight) in patients with nasopharyngeal cancer were observed with CC-486 (oral azacitidine) monotherapy. Overall, the study demonstrated that the drug is well tolerated in monotherapy as well as in combination with carboplatin or nab-paclitaxel (NCT02269943) [91]. A phase 3 study was conducted for the assessment of the platelet supportive effects of eltrombopag administered concomitantly with azacitidine. In comparison to azacytidine alone, the combination of eltrombopag and azacitidine led to the worsening of platelet recovery, with lower response rates. Moreover, increased progression to AML was evidenced [92] (NCT02158936). In another recently conducted phase 2 study, the combination of ruxolitinib and azacitidine was found to be safe. Improvement in bone marrow fibrosis coupled with significant spleen response rate was attained in patients with MF. The study design enrolled 46 patients and involved the administration of ruxolitinib twice per day continuously in 28-day cycles for the first 3 cycles followed by the addition of azacitidine (25 to 75 mg/m^2^, days 1–5) starting with cycle 4 [93] (NCT01787487).

Recently, Onureg (azacitidine 300 mg tablets, CC-486) was approved by US FDA for the continued treatment of adult patients in first remission with AML. The promising results of the AML-001 study (Phase 3 clinical trial) laid the foundation of FDA approval as statistically significant improvement in OS (10 months, median OS time 24.7 months, 95% CI: 18.7–30.5) compared to placebo (median OS time 14.8 months, 95% CI: 11.7–17.6) was attained by the use of onureg. [94] It is noteworthy to mention that a chemical stable analog of 5-Azacytidine, dihydro-5-azacytidine (DHAC), is also biologically active and is relatively less toxic [95, 96].

#### Decitabine

Decitabine, another nucleoside type DNMT inhibitor, is a desoxyribose analog of cytosine which only gets incorporated in DNA. Decitabine also leads to DNMT depletion and genome hypomethylation. Like, 5-Aza cytidine, decitabine has also been approved by FDA for the treatment of AML and MDS [74, 77]. In a phase II clinical investigation conducted to evaluate the efficacy of decitabine (IV, 15 mg/m^2^, 5 days–2 weeks) in patients with CML resistant to imatinib mesylate, 35 patients were enrolled (12 in chronic phase, 17 in accelerated phase, and six in blastic phase). The results of the study demonstrated complete hematologic responses in 12 patients (34%) and partial hematologic responses in seven patients (20%). Six patients exhibited major cytogenetic responses, and 10 demonstrated minor cytogenetic responses and the overall cytogenetic response rate observed was 46%. Major adverse effect evidenced was myelosuppression. Overall, it was concluded that decitabine is endowed with clinical activity in imatinib refractory CML [97]. A phase 2 clinical trial for the assessment of decitabine as maintenance therapy for younger adults with AML was conducted in anticipation that 1 year of maintenance therapy would lead to an improvement of disease-free survival for AML patients < 60 years, who as such were not responsive to allogeneic stem cell transplantation in first remission. The results of the study were not encouraging as the maintenance with decitabine did not exert any benefits [98] (NCT00416598). The dynamics of neoplastic cell clearance during decitabine treatment using quantitative monitoring of mutant alleles by pyrosequencing was investigated. The study results demonstrated that the drug was endowed with a noncytotoxic mechanism of action that leads to altered biology of the neoplastic clone and/or normal cells [99] (NCT00067808). A retrospective analysis was conducted to evaluate the response to decitabine in patients with advanced stage MDS. In the study, outcome of patients with baseline marrow blasts ≥ 20% and < 30% (refractory anaemia with Excess Blasts in Transformation—RAEB-t group) and < 20% (MDS group) were compared. A better duration of response was demonstrated by the patients with MDS (9.9 vs. 5 months; P = 0.024) and OS (16.6 vs. 9.0 months) in comparison to patients with RAEB-t [100] (NCT00043381, NCT00260065). A gene expression analysis to assess the gene expression patterns associated with response to decitabine was conducted in a multicenter phase II trial in older AML patients deemed unsuitable for induction chemotherapy. The results of the study indicated that the efficacy of decitabine is partly dependent on immunomodulatory effects [101] (NCT00866073).

In a Phase II study conducted with an aim to assess tosedostat in combination with cytarabine or decitabine in patients (newly diagnosed older) with AML or high‐risk MDS, 34 patients ≥ 60 years old were randomized and tosedostat (120 mg on days 1–21 or 180 mg continuously) was administered with decitabine (20 mg/m^2^/d) every 35 d. The study outcome indicates that combination of tosedostat and decitabine was tolerated well and resulted in a CR/CRi rate of > 50%. (NCT01567059) [102]. Recently, an inqovi (decitabine and cedazuridine) tablet for treatment of adult patients was approved by US FDA for the treatment of MDS and chronic myelomonocytic leukemia. The approval was attributed to the results of clinical trial that demonstrated similar drug concentrations between intravenous decitabine and inqovi. It was also observed that a considerable proportion of patients that were previously dependent on transfusions did not require the transfusions during an 8-week period. Moreover, intravenous decitabine displayed a similar safety profile to inqovi [103].

#### Guadecitabine

Guadecitabine, a next-generation hypomethylating agent, is a dinucleotide antimetabolite of a decitabine linked via phosphodiester bond to guanosine. Guadecitabine prolongs the exposure of tumor cells to the active metabolite, decitabine, leading to an enhanced uptake of decitabine into the DNA of rapidly dividing cancer cells. Guadecitabine also offers resistance to degradation by cytidine deaminase [104]. A study (Phase I/II) with an aim to determine the genomic and epigenomic predictors of response to guadecitabine in R/R AML was recently carried out. The study results indicated a 17% response rate to guadecitabine (2 CR, 3 CR with CRi or CR with CRp in the phase I component and 23% (14 CR, 9 CRi/CRp) in phase II. Peripheral blood blasts and haemoglobin were identified as predictors of response and cytogenetics, gene expression, RAS mutations, and haemoglobin as predictors of survival. [NCT01261312, [105]. In a phase 2 study evaluating the combination of guadecitabine with carbotaxol in heavily pretreated patients (n = 100 enrolled) with platinum-resistant recurrent ovarian cancer, promising activity was attained. No serious adverse events were observed in the study. Neutropenia (67%), leukopenia (25%) and anemia (14%) were evidenced as grade 3/4 events. The efficacy evaluation results were as follows: ORR (16%), DCR (37%), PFS (4.1 months), OS (11 months) [106]. In a phase 2 study conducted in patients with HCC, guadecitabine (45 mg/m^2^) administered on a 28-day cycle was well tolerated in subjects with HCC previously progressed on sorafenib. The study outcome revealed that potent global DNA demethylation (LINE-1) was observed in blood and tumor DNA. To add on, demethylation was seen in patients on promoter of TSG MZB1, which as such, is silenced in HCC [107] (NCT01752933). Recently, the efficacy and safety of guadecitabine was evaluated in phase III study (ASTRAL-1 study) in adults with previously untreated AML. The patients selected were ineligible for intensive induction chemotherapy. The study design involved the administration of guadecitabine, delivered (SC, 60 mg/m^2^/day for 5 days) in combination with either azacitidine (IV or SC 75 mg/m^2^/day, 7 days), decitabine (IV 20 mg/m^2^/day, 5 days) or low dose cytarabine (SC 20 mg bid, 10 days), administered in 28-day cycles. The results of this investigation revealed that primary end points of CR rate or OS were not met (NCT02348489) [108]. Recently, Astex and Otsuka announced the evaluation results of guadecitabine in phase 3 ASTRAL-2 and ASTRAL-3 studies in patients with previously treated AML and MDS or CML. It is disappointing to mention that the guadecitabine did not improve the OS and the study was unable to meet the primary end point [109].

### 5-Fluoro-2′-deoxycytidine (FdCyd)

5-Fluoro-2′-deoxycytidine represents another deoxyribonucleoside analog that undergoes phosphorylation and is capable of getting incorporated into DNA. The combination of FdCyd and the CD inhibitor tetrahydrouridine (THU) was evaluated in phase I study conducted in cynomolgus monkeys. The results of the investigation indicated that THU administration with FdCyd led to increase in the exposure to FdCyd and improved PO FdCyd bioavailability from < 1 to 24%. Moreover, THU and FdCyd concentrations achieved after PO administration were found to be associated with CD inhibition and hypomethylation, respectively [NCT00378807] [110]. In another phase I investigation of oral 5-fluoro-2′-deoxycytidine with oral THU in patients (N = 40) with advanced solid tumors, FdCyd was administered for 3 − 7 days q wk × 2 in 21-day cycles in combination with THU (administered, PO 30 min prior to Foci). The results of the study are as follows: MTD: FdCyd (160 mg) + THU (3000 mg), 1 × daily days 1 − 6 and 8 − 13, grade 4 toxicities: thrombocytopenia (1 pt), neutropenia (3 pts) and lymphopenia (3 pts), SD: 19 pts [111]. A phase I study was conducted to establish the pharmacokinetic and pharmacodynamics profile of FdCyd (IV) administered with THU (fixed dose − 350 mg/m^2^) in subjects with advanced cancer. The results of the study are as follows: MTD: Fdcyd (134 mg/m^2^) + THU (350 mg/m^2)^, days 1–5 and 8–12 every 4 weeks, Phase II dose determined − 100 mg/m^2^/day FdCyd with 350 mg/m^2^/day THU, good plasma exposures and the sustained PR was observed at 67 mg/m^2^/day [112] (NCT00378807). Recently, another study was carried out to evaluate the efficacy of 5-FdCyd in patients with advanced solid tumors. In the study, 93 patients were enrolled (29 breast, 21 head and neck cancer, 25 NSCLC, and 18 urothelial). The outcome of the study was not satisfactory as insufficient responses were achieved and only three PRs were attained. It is noteworthy to mention that the results were only promising in patients with urothelial carcinoma as the preliminary 4-month PFS rate of 42% was attained in the urothelial stratum. In 69% of the patients evaluable for clinical and CTC response, p16-expressing cytokeratin-positive CTCs were increased. Overall, the results observed in this study indicate exploration of FdCyd + THU in future is warranted in urothelial carcinoma [113].

#### Zebularine

Other than these FDA approved DNMT inhibitors, zebularine (4-Deoxyuridine, ribonucleoside analog), an oral DNA-demethylating drug has demonstrated stability in acidic environments as well as aqueous solutions. Despite being a potential DNMT inhibitor, its clinical translation has been hindered by the limited bioavalability in (< 7%) and primates (< 1%) along with high dose requirements in millimolar concentrations. [77, 114].

#### Non-nucleoside DNMT inhibitors

Risk of mutagenicity and genomic instability associated with the use of nucleoside DNMT inhibitors [75] has led to the initiation of numerous investigations with an aim of developing nonnucleoside analogs. Most of the non-nucleoside DNMT inhibitors developed so far is small molecule agents that directly target the catalytic sites rather than incorporating into DNA. This section presents a brief account of non-nucleoside inhibitors for natural and synthetic sources.

The sponge Pseudoceratina purpurea yields Psammaplin, a non-nucleoside based dual inhibitor of DNMT and HDAC [115]. A Polyphenol from green tea, EGCG ((-)-epigallocatechin-3-gallate reversibly demethylates methyl-DNA leading to the reactivation of multiple key genes (hMLH1, P16, and RA, in colon, esophageal, and prostate cancer cell lines) [116]. A polyphenolic compound, curcumin, has also been reported to induce global hypomethylation in MV4-11 leukemia cell lines possibly through covalently blocking of the catalytic thiolate of DNMT1, inhibiting DNA methylation [117]. Hydralazine and procainamide have demonstrated tumor suppressor reactivating and antitumor actions in breast cancer [118–120]. In a phase II study conducted to combat the issue of chemotherapy resistance in refractory solid tumors, addition of hydralazine and valproate to the same chemotherapy schedule that the patients were receiving, yielded clinical benefits in the selected population. [NCT00404508) [121]. A randomized phase III, epigenetic therapy with hydralazine valproate and chemotherapy in patients with advanced cervical cancer was also carried out. The study design involved the administration of hydralazine (182 mg—rapid acetylators, or 83 mg—slow acetylators along with valproate (30 mg/kg). The study was conducted in 36 patients and four PRs to CT (cisplatin topotecan) + HV (hydralazine valproate) and one in CT + PLA were achieved. SD in five (29%) and six (32%) patients was observed whereas eight (47%) and 12 (63%) showed progression (*P* = 0.27). Moreover, the study indicated substantial benefits in context of PFS [122] (NCT00532818). Other than these small molecule inhibitors, a second generation phosphorothioate antisense oligodeoxynucleotide, MG98 prevents DNMT1 mRNA translation effects and is under detailed preclinical studies and clinical stage investigations (phase I/II clinical trials) in solid tumors [123–125]. Another, small molecule inhibitor, RG-108 is reported to directly inhibit DNMT1 catalytic domain and block DNMTs without causing enzyme degradation [115, 126]. Disulfiram was also identified as a DNMTi as it was found to reduce global 5mC levels, as well as demethylate and reactivate the expression of epigenetically silenced TSGs [127]. SGI-1027, a quinolone based compound, exhibited inhibitory potential towards DNMT1, DNMT3A and DNMT3B, leading to demethylation and reactivation of TSGs [128]. Table 1 presents the clinical update of DNMT inhibitors undergoing clinical stage investigations.

**Table 1:** Clinical update of DNMT inhibitors

Drug	Clinical studies
5-Azacytidine	*Azacytidine is undergoing the below mentioned studies:*
	Low-risk MDS (Phase III, NCT01566695, status—active, not recruiting)
	R/R T-cell lymphoma (Phase III, NCT03703375, status, recruiting)
	AML with complete remission (Phase III, NCT01757535, active, not recruiting)
	MDS patients with excess blasts, progressing (Azacitidine + rigosertib, Phase III, NCT01928537, status—active, not recruiting)
	AML, MDS, CML (Azacitidine + HAG regimen, Phase III, NCT03873311, not yet recruiting)
	AML (Azacitidine + venetoclax, NCT02993523, Phase III, status—recruiting)
	MDS (Azacitidine + APR-246, NCT03745716, Phase III, status—recruiting)
	AML, MDS, CML (Azacitidine + pevonedistat, Phase III, NCT03268954, status—recruiting)
	AML with IDH1 mutation (Azacitidine + AG-120, Phase III, NCT03173248, status—recruiting)
	AML (Azacitidine vs fludarabine + cytarabine, phase III, NCT02319135, status—active, not recruiting)
	AML (Azacitidine + intensive chemotherapy, phase III, NCT03416179, status—recruiting)
	Head and neck squamous cell carcinoma (Phase II, NCT02178072, status—recruiting)
	Pancreatic cancer (Phase II, NCT01845805, status—recruiting)
	Solid tumors and hematological disorders (Phase II, NCT02494258, status—recruiting)
	AML (Azacitidine + venetoclax, Phase II, NCT03466294, NCT03573024, status—recruiting)
	MDS, CMML and AML relapsing after allo-HSCT (Azacitidine + lenalidomide + DLI 50, Phase II, NCT02472691, status—active, not recruiting)
	MDS with excess blasts 2 (Azacitidine + vosaroxin, Phase II, NCT03338348, status—ecruiting)
	Advanced solid tumors (Azacitidine + durvalumab, Phase II, NCT02811497, status—recruiting)
	High-risk MDS, AML (Azacitidine + durvalumab, NCT02775903, Phase II, status—active, not recruiting)
	AML with NPM1 mutation (Azacitidine + pembrolizumab, Phase II, NCT03769532, status—not yet recruiting)
	Pancreatic cancer (Azacitidine + pembrolizumab, NCT03264404, Phase II, status—recruiting)
	Metastatic melanoma (Azacitidine + pembrolizumab, Phase II, NCT02816021, status—recruiting)
	MDS (Azacitidine + pembrolizumab, Phase II, NCT03094637, status—recruiting)
	Chemorefractory metastatic colorectal cancer (Azacitidine + pembrolizumab, Phase II, NCT02260440, status—active, not recruiting)
	Advanced or metastatic non-small-cell lung cancer (Azacitidine + pembrolizumab, Phase II, NCT02546986, status—active, not recruiting)
	Platinum-resistant ovarian cancer (Azacitidine + pembrolizuma, Phase II, NCT02900560, status—recruiting)
	Prostate cancer (Azacitidine + ATRA, Phase II, NCT03572387, status—recruiting)
	Recurrent or refractory disease with IDH2 mutation (Azacitidine + enasidenib, Phase II, NCT03683433, status—recruiting)
	High-risk MDS with IDH2 mutation (Azacitidine + enasidenib, Phase II, NCT03383575, status—recruiting)
	R/R AML (Azacitidine + pevonedistat, Phase II, NCT03745352, status—not yet recruiting)
	High-risk MDS, AML, CML (Azacitidine + pevonedistat, Phase II, NCT02610777, status—active, not recruiting)
	AML without remission after allogeneic stem cell transplantation (Azacitidine + pevonedistat, Phase II, NCT03709576, status—recruiting)
	MDS, AML and CMML (Azacitidine + PF-04449913, Phase II, NCT02367456**,** status—recruiting)
	PTCL (Azacitidine + CHOP, phase II, NCT03542266, status—recruiting)
	Advanced non-small-cell lung cancer (Azacitidine + paclitaxel, phase II, NCT02250326, status**—**active, not recruiting)
	MDS (Azacitidine + pevonedistat, Phase II, NCT03238248, status—recruiting)
	Elderly patients with AML (Azacitidine + gemtuzumab ozogamicin, Phase II, NCT00658814, status—active, not recruiting)
	Refractory/relapsed AML (Azacitidine + ipilimumab + nivolumab, Phase II, NCT02397720, status—recruiting)
	MDS (Azacitidine + nivolumab + ipilimumab, Phase II, NCT02530463, status—recruiting)
	High-risk MDS, AML (Azacitidine + sirolimus, Phase II, NCT01869114, status—recruiting)
	R/R diffuse large B-cell lymphoma (Azacitidine + rituximab, Phase II, NCT03719989, status—not yet recruiting)
	R/R AML (Azacitidine + avelumab, Phase I/II, NCT02953561, status—recruiting)
	R/R AML, MDS (Azacitidine + quizartinib, phase I/II, NCT01892371, status—recruiting)
	AML, high-risk MDS (Azacitidine + cytarabine + tosedostat, phase I/II, NCT01636609, status—active, not recruiting)
	MDS (Azacitidine + sonidegib, Phase I, NCT02129101, status—active, not recruiting)
Decitabine (5-aza-2′deoxycytidine)-based trials	Decitabine is undergoing the below mentioned studies
	R/R diffuse large B-cell lymphoma (Phase IV, NCT03579082, status—recruiting**)**
	R/R T lymphoblastic lymphoma (Decitabine, Phase IV, NCT03558412, status—recruiting)
	PTCL (Decitabine + CHOP, Phase III, NCT03553537, status—not yet recruiting)
	AML with TP53 mutation (Decitabine, phase II, NCT03063203, status—recruiting)
	AML (Decitabine + clofarabine, Phase II, NCT02085408, active, status—not recruiting)
	AML (Decitabine + bortezomib, phase II, NCT01420926, status—active, not recruiting)
	AML (Decitabine + cytarabine + daunorubicin hydrochloride, Phase II, NCT01627041, status—active, not recruiting)
	Relapsed FLT3-ITD-mutated AML, MDS (Decitabine + quizartinib, Phase I/II, NCT03661307, status—recruiting)
	AML (Decitabine + ruxolitinib, Phosphate, Phase I/II, NCT02257138, status—recruiting)
	Metastatic castration-resistant prostate cancer (Decitabine + enzalutamide, Phase I/II, NCT03709550, status—not yet recruiting)
Guadecitabine (SGI-110)-based trials	Guadecitabine is currently being assessed in ASTRAL-2 (Phase III, R/R AML, NCT02920008) and ASTRAL-3 (phase III, MDSs (MDS) or chronic myelomonocytic leukemia, NCT02907359)
	Guadecitabine is undergoing the below mentioned studies: MDS, CMML (Guadecitabine, NCT02907359, Phase III, status—recruiting)
	Philadelphia-negative MDS (Guadecitabine, Phase II, NCT03075826, status—recruiting)
	High-risk MDS (Guadecitabine, NCT02131597, Phase II, status—recruiting)
	MDS relapsing post AlloSCT (Guadecitabine + DLI, NCT02684162, Phase II, status—recruiting)
	Pembrolizumab, Phase II, NCT02901899, status—recruiting)
	Metastatic colorectal cancer (Guadecitabine + irinotecan, Phase II, NCT01896856, status—active, not recruiting)
	Refractory or resistant urothelial carcinoma (Guadecitabine + atezolizumab, Phase II, NCT03179943, status—recruiting)
	Refractory metastatic colorectal cancer (Guadecitabine + nivolumab, Phase I/II, NCT03576963, status—Not yet recruiting)
	Recurrent ovarian, primary peritoneal, or fallopian tube cancer (Guadecitabine + )
	Advanced kidney cancer (Guadecitabine + durvalumab, Phase I/II, NCT03308396, status—recruiting)
	Advanced MDS CMML (Guadecitabine + atezolizumab, Phase I/II, NCT0293536 status—recruiting)
	Recurrent ovarian, fallopian tube, or primary peritoneal cancer (Guadecitabine + CDX-1401 Vaccine + atezolizumab, Phase I/II, NCT03206047, status—recruiting)
	AML, MDS (Guadecitabine + DLI, Phase I, NCT03454984, status—not yet recruiting)
Hydralazine-based trials	Hydralazine is undergoing the below mentioned studies:
	Ovarian cancer (Hydralazine + valproate, Phase III, NCT00533299, status—N/A)
	Cervical cancer (Hydralazine + valproate, Phase III, NCT00532818, status—N/A)
	Recurrent-persistent (cervical cancer, Hydralazine + valproate, Phase III, NCT02446652, status—N/A)

### EZH2 inhibitors

**Fig. 3:**
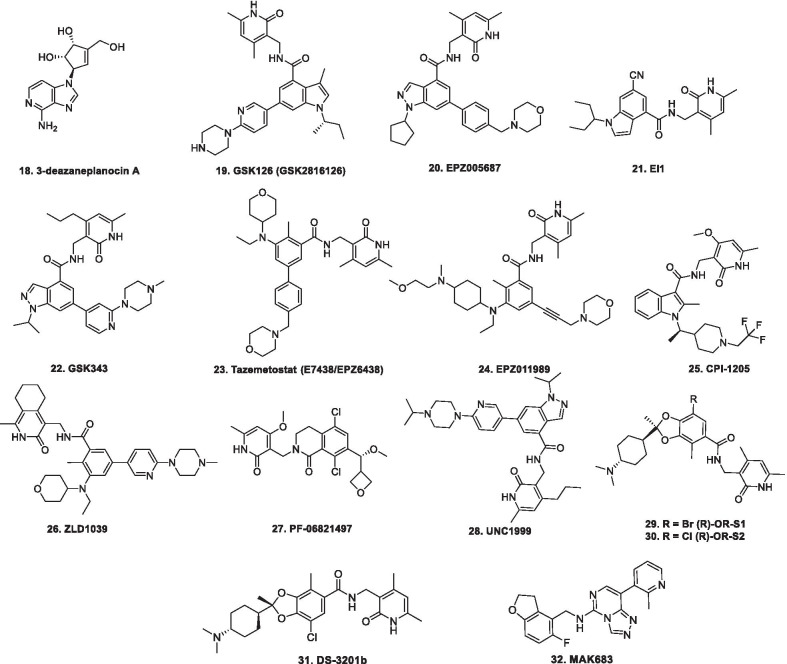
EZH2 inhibitors

EZH2, a crux subunit of the PRC2, is a HMT enzyme responsible for methylating lysine 27 (mono-, di- and trimethylation) in histone H3 (H3K27). H3K27me3 is more frequently interlinked with transcriptional repression, and it is a significant epigenetic phenomenon during tissue development and stem cell fate determination. Specifically, functioning of EZH2 in biological processes occurs through 3 types of mechanism viz. PRC2-dependent H3K27 methylation, PRC2-dependent non-histone protein methylation, and PRC2-independent gene transactivation [129–136]. As such, EZH2 works as a master regulator of cell cycle progression [137], autophagy, and apoptosis [138], promotes DNA damage repair and inhibits cellular senescence [139]. In view of the aforementioned notions, it is evident that EZH2 plays an important role in cell lineage determination and relative signalling pathways. The enzyme has been found to be overexpressed in wide varieties of cancer, such as prostate, liver, gastric, breast, bladder, lung, and pancreatic cancers [129–135] with literature precedents ascertaining the role of EZH2 in augmenting the development and progression of cancer. In this context, EZH2 targeting therapies, at present, have garnered significant attention for the treatment of many types of cancer. Till now, tazemetostat stands as the only approved EZH2 inhibitor for advanced epithelioid sarcoma, however, various EZH2 inhibitors are being evaluated at preclinical and clinical stages. The details of selected important EZH2 inhibitors are mentioned in Table 2 and the structures are shown in Fig. 3. It is noteworthy to mention that a homolog of EZH2, EZH1, is present in a non-canonical PRC2 complex. As such, EZH1 complements EZH2 in mediating H3K27 methylation and is also endowed with HMT activity. In light of this disclosure, it is highly anticipated that simultaneous inhibition of EZH1/EZH2 that can be attained via dual EZH1/EZH2 inhibitors might exert potent anticancer effects. A brief mention of a dual EZH1/EZH2 inhibitor is also included in Table 2 [140].

**Table 2:** Update of EZH2 inhibitors

Compound	Details
3-Deazaneplanocin A	First EZH2 inhibitor that indirectly inhibits EZH2 via S-adenosyl-l-homocysteine increase and exerts direct repression of S-adenosyl-l-methionine-dependent histone methyltransferase activity [141]
GSK126 (GSK2816126)	A highly selective and potent inhibitor of EZH2 [142] In a phase 1 clinical trial of GSK126 conducted in patients of advanced hematologic and solid tumors, escalating doses of GSK126 (50–3000 mg, twice weekly as an intravenous solution for 28 days (3 weeks on/1 week off) were administered to 41 participants (21 solid tumors, 20 lymphoma). The outcome of the study did not demonstrate sufficient evidence of clinical activity [142]
EPZ005687	EZH2 inhibitor that possesses high affinity as well as selectivity for EZH2, however is endowed with unfavourable pharmacokinetic properties. [143]
EI1	A highly selective SAM-competitive inhibitor of EZH2 Inhibits the growth of DLBCL cells carrying Y641 mutations. [144]
GSK343	SAM-competitive inhibitors of EZH2. [145]
	The drug can suppress the levels of histone H3K27me3 and cause inhibition of EZH2 activity in breast and prostate cancer cells [145]
	The use of GSK 343 in in vivo studies might be hindered by the evidenced high clearance in rat PK studies [145]
	In a preclinical study, the antitumor effects of GSK343 on glioma cells were evaluated in vitro and in vivo*.* The results of the study highlighted the potential of GSK343 to reduce the proliferation, attenuate cell motility and reverse epithelial-mesenchymal transition in U87 and LN229 glioma cells. It was also observed that GSK343 suppressed the stemness of cell lines and patient derived glioma stem cells. Moreover, Histone H3K27 methylation was inhibited by GSK343 inhibited histone H3K27 methylation. Cumulatively, the results portended that GSK343 could be emerge as a potent weapon against the glioma. [146]
Tazemetostat (E7438/EPZ6438)	An orally administered, first-in-class small molecule EZH2 inhibitor [147–152]. The discovery of tazemetostat involved extensive structural engineering attempts on a bicyclic ring bearing EZH2 inhibitor (initial hit compound). As a result of the attempts centred on identifying structural prerequisites for amplifying the EZH2 inhibition, it was found that disconnecting the five-membered ring of the bicyclic core increased the potency and rendered an additional site that could be exploited for enhancing the polarity of the adducts, thereby imparting ideal physicochemical properties to the compounds. Overall, an amide tethered dimethyl substituted pyridone ring on a THP decorated aniline was found to be the key structural feature for exerting EZH2 inhibition and the installation of the benzyl morpholine ring was deduced to be instrumental in improving the physicochemical properties of the constructs. [147]
	Tazemetostat is endowed with improved potency and favourable pharmacokinetic properties in comparison to EPZ005687. [148]
	Accelerated approval was granted by US FDA to tazemetostat on 23^rd^ January for patients with metastatic or locally advanced epithelioid sarcoma [149]
	A phase 2 clinical study of tazemetostat in patients with R/R B-cell NHL is ongoing. The interim assessment indicates that tazemetostat is endowed with preliminary clinical activity in pts with R/R DLBCL and FL. The drug was particularly found to be beneficial in subjects with tumours bearing activating EZH2 mutations. Moreover, the drug was found be safe. The results (interim efficacy results attained from 149 patients) are as follows:
	1. The ORR (CR + PR)—40% in pts with DLBCL with EZH2 mutations (N = 10),
	2. ORR—18% in pts with DLBCL with wild type (wt) EZH2 (N = 85),
	3. ORR—63% in FL pts with EZH2 mutations (N = 8) 28% in FL pts with wt EZH2 (N = 46). [25] (NCT03456726)
	In another phase 2 study, tazemetostat as single agent was evaluated in adult patients with R/R MM with BAP1 inactivation. In the study, 800 mg (po BID) of tazemetostat was administered. The results of the study are as follows:
	1.N = 74 patients**,** 5 pts had dose reductions due to AEs. The frequently observed AEs were Fatigue (32%), decreased appetite (28%), dyspnea (28%), and nausea (27%)
	2.Disease control was achieved in 31 pts (51%) at 12 weeks
	3.Sustained disease control was attained in 15 pts (25%) at 24 weeks, 5 of whom are ongoing 4.Overall, tazemetostat exhibited safety, efficacy as well as tolerability in patients with MM. (NCT02860286) [151]
	A Phase 1 study of tazemetostat in R/R B-cell NHL in patients with advanced solid tumours was conducted. In the study, tazemetostat was administered to 64 patients (21 with B-cell non-Hodgkin lymphoma, and 43 with advanced solid tumours). The RP2D was identified as 800 mg twice daily. Durable ORs were achieved in 8 patients out of 21 patients with B-cell NHL, while only two patients out of 43 patients with solid tumours displayed durable objective responses. Overall, the drug was found to be safe and clinically active in patients with refractory B-cell NHL and advanced solid tumours. [152] (NCT01897571)
	Tazemetostat is undergoing the below mentioned studies:
	R/R FL (Tazemetostat in combination with Lenalidomide Plus Rituximab, Phase 3, NCT04224493, status—not yet recruiting)
	Advanced Epithelioid Sarcoma (Tazemetostat in Combination with Doxorubicin, Phase 3, NCT04204941)
	R/R B-cell Non-Hodgkin's Lymphoma (Tazemetostat, Phase 2, NCT03456726, status—active, not recruiting)
	Recurrent or Persistent Endometrioid or Clear Cell Carcinoma of the Ovary, and Recurrent or Persistent Endometrioid Endometrial Adenocarcinoma (Tazemetostat, Phase 2, NCT03348631, status—suspended)
	Tumors Harboring Alterations in EZH2 or Members of the SWI/SNF Complex (Tazemetostat, Phase 2, NCT03213665, status—recruiting)
	INI1-Negative Tumors or R/R Synovial Sarcoma (Tazemetostat, Phase 2, NCT02601950, status—recruiting)
	Newly Diagnosed Diffuse Large B Cell Lymphoma (DLBCL) (Tazemetostat, Phase 1/2, NCT02889523, status—suspended)
	Advanced Solid Tumors or with B-cell Lymphoma (Tazemetostat in combination with prednisolone, phase1/2, NCT04179864, status –active, not recruiting)
	B-cell Lymphoma or Advanced Solid Tumors (Tazemetostat, Phase 1, NCT03028103, status—active, not recruiting)
	Advanced Malignancies (Tazemetostat, Phase 1, NCT04241835, status—recruiting)
	R/R B-cell Non-Hodgkin's Lymphoma (Tazemetostat, Phase 1, NCT03009344, status—active, not recruiting,)
	Tazemetostat Rollover Study (TRuST) (Tazemetostat, Phase 1, NCT02875548, status—recruiting)
	R/R INI1-Negative Tumors or Synovial Sarcoma (Tazemetostat, phase 1, NCT02601937, status—recruiting)
EPZ011989	A selective and orally bioavailable EZH2 inhibitor
	Exerts significant tumor growth inhibition in mouse xenograft model of human B cell lymphoma [153]
CPI-169	An indole based EZH2 inhibitor
	Demonstrated substantial antiproliferative activity and pharmacodynamics (PD) target engagement in a mouse xenograft model of a KARPAS-422 lymphoma
	Suffers from the issue of limited oral bioavailability [154]
CPI-1205	Optimized from the structural engineering attempts on CPI-169, CP1-1205 is also an indole based small molecule inhibitors of EZH2 [155]
	In a phase 1 study of CPI-1205 in patients with B-Cell lymphomas, CPI-1205 was administered orally twice daily (BID, in 28-day cycles) in 4 dose cohorts. The results of the study are mentioned below:
	n = 32 pts, drug related AEs were mostly grade 2 and lower, treatment-related AEs in ≥ 5% pts of any grade were nausea, diarrhea, anemia and fatigue, TRAEs ≥ grade 3 were observed in 7 patients, DLTs were not observed, CR was observed in 1 patient and SD was observed in 5 pts. CPI-1205 was found to be endowed with short half-life. Overall, the drug was found to be well tolerated with manageable toxicities. Antitumor activity was observed along with target engagement that was evaluated by assessing the H3K27me3 reduction by IHC in skin and lymphoma tissue. [156]
	CPI-1205 is presently undergoing the below mentioned studies
	mCRPC (CPI-1205 + enzalutamide or abiraterone/prednisone, Phase IB/II NCT03480646 (ProSTAR), status—active, not recruiting)
	Advanced solid tumor (phase 1/2 clinical trial, NCT03525795)
CPI-0209	Second-generation EZH2 inhibitor endowed with higher anticancer potency in comparison to first-generation EZH2 inhibitors as per the results of preclinical studies conducted in multiple cancer types
	The drug is anticipated to achieve comprehensive target coverage via extended on-target residence time [155]
	The drug is undergoing phase 1 clinical trials in patients with advanced solid tumors (CPI-02029-monotherapy and combination therapy, Phase 1/2, NCT04104776, status—recruiting)
SHR2554/SHR3680 Structure undisclosed	An orally available EZH2 inhibitor
	SHR2554 is undergoing the below mentioned clinical studies
	mCRPC (SHR2554 in combination with SHR3680 (Anti androgen, Phase 1/2, NCT03741712)
	Advanced or metastatic solid tumors and R/R B-cell lymphomas (SHR2554 in combination with Anti-PD-L1/TGFβ Antibody SHR1701, Phase1/2, NCT04407741, status—not yet recruiting)
	Phase 1 clinical investigation in patients with Refractory mature lymphoid neoplasms. (status—recruiting, NCT03603951)
ZLD1039	A highly selective, and orally bioavailable inhibitor of EZH2
	Exerts inhibition of breast tumor growth and metastasis in mice [157]
PF-06821497	A small molecule potent and selective inhibitor of EZH2
	It is active against both wild-type (wt) as well as mutant EZH2 [158]
	PF-06821497 is currently under evaluation in a phase 1 clinical trial in patients with R/R SCLC, CRPC, FL and DLBCL (NCT03460977, status—recruiting)
UNC1999	Oral SAM competitive inhibitor of wild-type (wt) and Y641 mutant EZH2 as well as EZH1 [159]
	Preclinical investigations have revealed that it effectively inhibits the growth of MLL rearranged leukemia in mice [159]
(R)-OR-S1 and (R)-OR-S2	OR-S1 and OR-S2 are S-adenosylmethionine (SAM)-competitive and highly selective
	EZH1/2 dual inhibitors
	Exhibit greater antitumor efficacy than selective EZH2 selective inhibitor against
	KARPAS-422 cells harboring a GOF mutation in EZH2 [160]
DS-3201b	A potent inhibitor of EZH1 and EZH2
	In preclinical studies, DS-320Ib has demonstrated antitumor activity against various hematological malignancies
	In a dose escalation phase 1 study in patients with R/R Non Hodgkin Lymphomas, the efficacy of DS-3201b (administered orally once daily (QD) over 28-days (1 cycle) continuously until disease progression) was evaluated. Overall, the results of the study demonstrated that DS-3201b is endowed with clinical activity and exhibited promise to be an orally available, therapeutic option for B-cell and T-cell lymphomas. Specifically, 1 CR, 7 PR and 5 SD of 15 patients (ORR = 53%) was observed. For T-cell lymphoma, ORR was 80% (1 CR and 3 PR out of 5 patients). (NCT02732275) [161, 162]
MAK683	An inhibitor of EED protein and allosteric inhibitor of PRC2
	EED-EZH2 protein–protein interaction (PPI) disruption leads to loss of H3K27me3-stimulated PRC2 activity and prevents H3K27 trimethylation, which ultimately leads to decreased tumor cell proliferation in EZH2-mutated and PRC2-dependent cancer cells. [163, 164]
	MAK683 is undergoing the below mentioned clinical study:
	DLBCL, nasopharyngeal carcinoma, gastric cancer, ovarian cancer, prostate cancer, and sarcoma (Phase 1/2 clinical trial, NCT02900651 status—recruiting) [163, 164]

The results covered in this Table 2 indicates that EZH2, at present, is considered as potential target for the design of cancer therapeutics and many EZH2 inhibitors are under development and evaluation in clinical trials. Other than the promising results of EZH2 inhibitors in monotherapy, combination of EZH2 inhibitors with immunotherapy or chemotherapy has also demonstrated synergism and is likely to be the futuristic strategy to extract therapeutic benefits for EZH2 inhibition. Researchers are also employing rational approaches for accomplishing new EZH2 inhibitors that can display high efficacy and low selectivity.

### DOTIL inhibitors

**Fig. 4:**
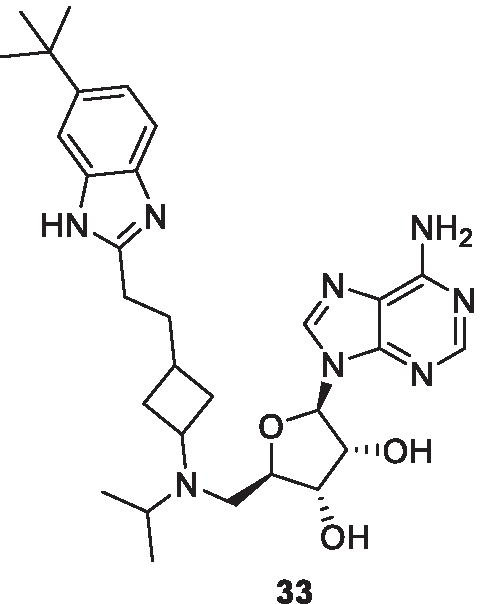
Pinometostat

H3K79 (Methylation of histone 3 at lysine 79) (H3K79) is one of the main mechanisms involved in gene expression. HMT DOT1L targets the histone H3lysine 79 (H3K79) residue for mono-, di- and tri-methylation. As such, DOT1L has a critical role in the regulation of gene transcription, development, cell cycle progression and DNA damage repair. Specifically, DOT1L leads to enhanced H3K79 methylation, methylation of open chromatin, downstream oncogenes overexpression and leukemogenesis via interaction with mixed lineage leukemia [165]. Studies have revealed that changes in normal expression levels of DOT1L have been found in prostate, breast, and ovarian cancer. In addition, H3K79me levels are elevated in AML patients bearing MLL rearrangements [166]. In light of the aforementioned, attention has been paid towards the development of small molecule DOT1L inhibitors and accordingly pinometostat (Fig. 4), a potent and selective small molecule DOT1L inhibitor endowed with subnanomolar affinity for DOT1L and > 37 000-fold selectivity against towards HMT has been investigated at the clinical stage [167–170]. The results of the preclinical studies indicated that it selectively inhibits intracellular H3K79 methylation in a concentration- and time-dependent manner. Pinometostat demonstrated activity against leukemia involving MLL-r in in vivo rodent xenograft studies [171–173]. Subsequently, a phase 1 study of pinometostat (dose escalation study) was performed in subjects with R/R MLL-r leukemia. The study design involved the administration of pinometostat via continuous intravenous infusion until disease progression or unacceptable toxicity. A total of 18 patients were enrolled for the investigation, 9 of them received 70 mg/m^2^/day of pinometostat, 7 patients were dosed at 90 mg/m^2^/day and the 2 patients were dosed at 45 mg/m^2^/day. The results of the study revealed that DLTs were observed which included hypocalcemia; hypophosphatemia; apnea, elevated transaminase, drug related AEs: anemia; thrombocytopenia; neutropenia; leukopenia; rash; lymphopenia; ALT elevation; nausea; vomiting. The drug demonstrated an acceptable safety profile and RP2D was determined as 70 mg/m^2^ CIV in children > 1 yr. As such, no objective responses were observed. (NCT02141828) [174]. In another phase 1 study, evaluating the efficacy of pinometostat (EPZ-5676) administered as continuous intravenous infusion in patients with MLL-r leukemia (adults). 51 patients were enrolled and CR was observed in 2 patients. Nausea, constipation, febrile neutropenia (grade 1 & 2) were observed as AEs. Overall, the results demonstrated that pinometostat was safe and endowed with modest efficacy in monotherapy. MTD was not determined in the study (NCT01684150). [175]

Other than the completed studies mentioned above, pinometostat is also undergoing clinical evaluation in combination with azacytidine in subjects with R/R, or newly diagnosed AML leukemia with 11q23 rearrangement (Phase 1/2 Studies, NCT03701295, status recruiting) and a Phase 1b/2 investigation in combination with standard induction chemotherapy in patients with newly diagnosed AML with MLL rearrangement (NCT03724084, status – recruiting).

### HDAC inhibitors

It is well known that post translational modifications of histones are involved in cancer development and progression via modulation of gene transcription, chromatin remodeling and nuclear architecture. Tightly controlled by the opposing activities of HATs and HDACs, histone acetylation is a well explored post translational modification. As such, HDACs removes the acetyl groups on N-terminal lysines of the histone proteins, thereby inducing histone hypoacetylation that leads to loosening of the chromatin structure ultimately facilitating the transcriptional process [176–186].

Eighteen HDACs in humans are categorized in to two families based on their catalytic mechanisms. Out of the 18, 11 are zinc dependent metalloenzymes (HDAC1-11) that catalyse the hydrolysis of amide bond using water as a nucleophile while the other 7 are sirtuins (1–7) that employ NAD + as a cofactor and transfer the acyl group to the ribose sugar (C2 position). The 18 human HDACs are further delved in to four classes. Class I (HDAC1, HDAC2, HDAC3 and HDAC8) Class IIa (HDAC4, HDAC5, HDAC7 and HDAC9) Class IIb (HDAC6 and HDAC10) Class III (sirtuins 1–7); Class IV (HDAC11) [176, 187–189]. Numerous studies have revealed that class I, II and IV are aberrantly expressed in diverse malignancies that majorly include solid and hematological tumors. Owing to this, HDACs as drug targets in cancer have been exhaustively explored and HDAC inhibitors are considered to the key for epigenetic cancer therapy owing to their ability to induce relief of transcriptional repression in various leukemias [177].

Structural fabrication of HDAC inhibitors includes a cap group, linker part and a Zn-binding group and HDAC inhibitors are generally categorized in to two structural classes on the basis of the zinc binding group: Hydroxamic acids and the aminoanilides. SAHA [68], Belinostat (PXD101) and Panobinostat (LBH589) [70] represent the US FDA approved hydroxamic acid type HDAC inhibitors and mocetinosat [190], MS-275 [191] and chidamide [72] are the representative examples of N-(2-Aminophenyl)benzamide binding unit containing HDAC inhibitors.

In 2006, suberoylanilide hydroxamic acid (SAHA) became the first HDAC inhibitor to get FDA approval for the treatment of CTCL. Spurred by the success of SAHA, several HDAC inhibitors with linear methylene chains have progressed to clinical stage investigation viz tefinostat [192], CG200745 [193], ricolinostat [194], citarinostat [195], CUDC-101 [196] and tinostamustine [197]. Another HDAC inhibitor, romidepsin [67] belonging to the family of depsipeptide natural products was approved for CTCL. Sipruchostatin, also belonging to the class of natural product depsipeptide is currently undergoing phase 1 clinical investigation for the treatment of solid tumors [198, 199]. Belinostat (PXD-101) that bears a more rigid alkenyl hydroxamic acid is the third HDAC inhibitor to be approved by US FDA in 2014 [69] for the treatment of PTCL. Another alkenyl type small molecule HDAC inhibitor, Panobinostat, received FDA approval in 2015 for the treatment of patients with multiple myeloma [70]. The FDA approvals of belinostat and panobinostat opened an avenue for the exploration of alkenyl type drug candidates and subsequently, resminostat [199–202] and pracinostat [203–205] were identified that are now undergoing clinical stage investigation. Givinostat [206–208], abexinostat [209, 210], AR-42 [211, 212] and bisthianostat [213, 214] represents the chemically related compounds bearing a more rigid phenylhydroxamic acid while quisinostat [215–217], nanatinostat [218, 219] and fimepinostat [220] containing a more polar pyrimidinyl heteroaromatic hydroxamic acid have also demonstrated substantial efficacy.

Ortho-aminoanilides constitute the other class of synthetic HDAC inhibitors that exhibit bidentate coordination of the carbonyl oxygen and aniline nitrogen with the active site zinc cation. Ortho-amino anilides are weak metal binders than hydroxamic acids and exhibit unusual kinetics along with slow and tight binding to HDACs. Tacedinaline [221], etinostat [222, 223], mocetinostat [224, 225], tucidinostat [226, 227], domatinostat [228] and CXD1 [229] represents the clinical candidates from this class of synthetic HDAC inhibitors. Among the benzamides, only tucidinostat (chidamide) stands as the approved benzamide (CFDA approved) for the treatment of patients with recurrent or refractory PTCL [226].

Some of the HDAC inhibitors such as CKD-504, 506, CS3003, HG116, KA2507, OK-179 (undisclosed structures) are also undergoing phase I clinical investigation. All of them belongs to the category of selective HDAC6 inhibitors except OKI-179 which is a synthetic analogue of largazole, isolated from a marine cyanobacterium of the genus Symploca, (selective Class I HDAC inhibitor) [224].

**Fig. 5:**
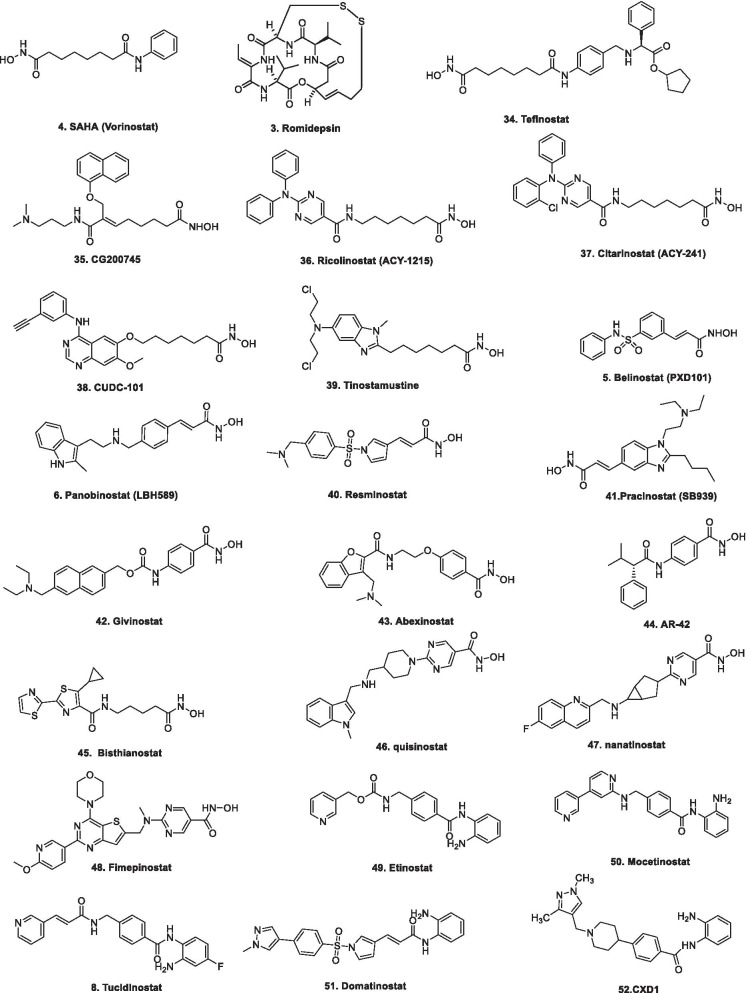
HDAC inhibitors

A clinical/preclinical update of the HDAC inhibitors is presented in Table 3 and the structure of the HDAC inhibitors are shown in Fig. 5.

**Table 3:** Update of HDAC inhibitors

Compound	Details
SAHA (Vorinostat) [11, 19]	Pan HDAC inhibitor
	FDA approved (CTCL)
	Developed by Merck
	Currently, SAHA is undergoing Phase 2 clinical trial in combination with pembrolimuzab and tamoxifen in patients with estrogen receptor positive breast cancer. (NCT04190056)
Romidepsin [67]	Developed by Bristol Myers Squibb
	Class 1 selective HDAC inhibitor
	FDA approved (CTCL, PTCL)
Tefinostat [192]	Developed by GlaxoSmithKline
	Monocyte/macrophage-targeted HDAC inhibitor
	A phase 1/2 study of tefinostat has been completed in patients of HCC. The results have not been published yet (NCT02759601)
	The phase 1 investigation of tefinostat (administered orally, once daily, n = 18, dose escalation—20–640 mg) in patients with R/R haematological diseases was conducted. Monocyte‐targeted increases in protein acetylation were evidenced as a result of flow cytometric assays. Maximum tolerated dose (MTD) was not identified. Grade 1/2 adverse events were observed that included nausea, anorexia, fatigue, constipation, rash and increased blood creatinine. A bone marrow response was observed in a patient with chronic monomyelocytic leukaemia. Moreover, a decrease in bone marrow blasts (50%) and clearance of peripheral blasts was observed in AML. Overall, the outcome of the study demonstrated that tefinostat was endowed with efficacy (NCT 00,820,508). [192]
CG200745 [193]	CG200745 is an intravenous hydroxamate-based pan-HDAC inhibitor
	To determine the MTD, safety and efficacy of CG200745 in subjects with MDS was completed (NCT02737462), however, the results have not published yet
	A combination study (Phase 1/2) of CG200745 with gemcitabine and erlotinib in patients with advanced pancreatic cancer has also been completed.(results not published, NCT02737228)
Ricolinostat (ACY-1215) [230]	Developed by Regenacy Pharmaceuticals
	First-in-class selective HDAC6 inhibitor
	Ricolinostat demonstrated efficacy in patients (> 250) with haematologic cancer. [230]
	In a combination study (with bortezomib and dexamethasone) in patients with R/R multiple myeloma, RP2D of ricolinostat was determined to be 160 mg daily. Moreover, the combination was found to be safe, well tolerated, and active. [231]
	A study (dose escalation, Phase 1b/2) was initiated to evaluate the combination of ACY-1215 with pomalidomide and low-dose dexamethasone in subjects with R/R multiple myeloma. (NCT01997840, status – active, not recruiting)
	In a phase 1b study, ricolinostat was administered to patients daily for 21 days of each 28-day cycle with nab-paclitaxel 100 mg/m^2^ on days 1, 8, and 15. The results of the study demonstrated that ricolinostat (240 mg qd) with nab-paclitaxel was safe and tolerable. In addition, majority of the patients demonstrated SD and 1 with PR. Moreover, clinical activity was also observed [NCT02632071]
Citarinostat (ACY-241)	A selective HDAC6 inhibitor Currently being investigated for the treatment of myeloma, melanoma, and NSCLC [195] A Phase 1a/b clinical investigation (ACE-MM-200) to evaluate the safety and efficacy of citarinostat alone and in combination (pomalidomide and dexamethasone) is currently ongoing in subjects (n = 85 patients) with R/R multiple myeloma. The initial results demonstrate that the drug was well tolerated (both alone and in combination) and was also found to be endowed with clinical activity (NCT02400242) Earlier, a dose escalation study to evaluate the efficacy of citarinostat in combination with paclitaxel in patients (n = 20) who failed to respond to previous treatment with advanced solid tumors revealed that the combination of citarinostat and paclitaxel is safe and demonstrated potential in heavily pretreated patients (NCT02551185) At present, a phase 1 clinical trial is currently enrolling patients to assess the efficacy of combination of citarinostat with PVX-410 and lenalidomide (NCT02886065)
CUDC-101	Quinozoline based small-molecule inhibitor (multi-targeted inhibitor) of EGFR, HER2, class I and class II HDACs [196] In a phase I study, escalating doses (75–300 mg/m^2^/day) of CUDC-101 was administered (1-h i.v. infusion for 5 consecutive days every 2 weeks) to 25 patients with advanced solid tumors. The results of the study indicated that CUDC-101 demonstrated clinical activity and was well tolerated. A dose of 275 mg/m^2^ was determined as MTD (NCT00728793). [196] The results of the phase I study revealed that the combination of CUDC-101, cisplatin, and radiation were feasible in head and neck squamous cell carcinoma. The study involved the intravenous administration of CUDC-101 for three times in a week followed by concurrent administration of cisplatin (100 mg/m^2^ every 3 weeks) and external beam radiation (70 Gy to gross disease) over 7 weeks. MTD was determined in the study, however, owing to DLT-independent discontinuation of CUDC-101, the results indicate that a change in the schedules or routes of administration is required (NCT01384799). [232]
Tinostamustine [197]	It is an alkylating HDAC inhibitor Chemically, it is composed by the fusion of alkylating agent bendamustine with SAHA In a phase 1 study in patients with advanced solid tumors, 60 mg/m^2^ tinostamustine was administered to the first cohort of patients followed by administration of maximum dose of 100 mg/m^2^ to the ascending 6 cohorts. A total of 22 patients were enrolled in the study. All the patients experienced ≥ 1 castration-resistant prostate cancer TAEs. Clinically significant QTC prolongation event was evidenced in only 1 patient. Overall, nostamustine demonstrated some efficacy and was well tolerated. [233] Tinostamustine is currently undergoing the below mentioned clinical stage investigations: Advanced melanoma (in combination with nivolumab, phase 1, NCT03903458, status – recruiting) R/R Hematologic Malignancies (Phase 1, NCT02576496, recruiting) Newly Diagnosed MGMT-Promoter unmethylated glioblastoma (phase 1, NCT03452930, recruiting)
Belinostat (PXD101) [69]	Developed by Onxeo, Spectrum FDA approved pan HDAC inhibitor for PTLC
Panobinostat (LBH589) [70]	Developed by Novartis Pan HDAC inhibitor approved for the treatment of multiple myeloma The combination of Panobinostat with azacitidine was evaluated in a phase 1b/2b multicenter study conducted in adults with MDS, CMML or AML. The results of the study led to the identification of the RP2D as PAN 30 mg plus AZA 75 mg/m^2^. [234] Panabinostat is undergoing the below mentioned clinical stage investigation: High risk AML and MDS (Phase 3, NCT04326764, recruiting) R/R Multiple Myeloma (Combination of Panobinostat and Carfilzomi, Phase 1/2, NCT01496118, status—active, not recruiting) Diffuse intrinsic pontine glioma (Phase 1, NCT02717455 recruiting)
Resminostat	An oral hydroxamate-type inhibitor of class I, IIB, and IV HDAC [200–202] A phase I/II investigation of resminostat in combination with sorafenib in patients with HCC was conducted. The study design involved the administration of sorafenib (400 mg, bid) in both phase I and II and administration of resminostat on days 1 to 5 every 14 days (dose escalation was carried in phase I from 400 mg/day to 600 mg/day). Patients were randomly subjected to sorafenib monotherapy or sorafenib/resminostat combination therapy (1:1 ratio) in phase 2 studies. In phase 1 study (n = 9 enrolled) grade 3–4 toxicities such as G4 thrombocytopenia was observed at the dose of 600 mg/day. Thus, 400 mg/day was determined as RP2D for Phase II studies. In phase II study, 170 pts were enrolled and the results demonstrated a median time to progression of 2.8 months in the combination and control arm. Significant difference was not observed in the median OS (NCT02400788) [199] Resminostat was also tested in R/R HL in a phase 2 study and the results demonstrated clear OR in R/R HL patients. Moreover, the drug was found to be endowed with excellent safety profiles in heavily pre-treated patient population (NCT01037478) [200] In a phase I/II study conducted in japanese patients with stage IIIB/IV or recurrent NSCLC and prior platinum-based chemotherapy, no DLT was observed in phase I part and the recommended dose was determined to be 600 mg/day with 75 mg/m^2^ of docetaxel. The results of the phase 2 part demonstrated Median PFS (95% CI—4.2 (2.8–5.7) months with docetaxel group and 4.1 (1.5–5.4) months with docetaxel—resminostat group. Overall, docetaxel resminostat therapy did not improve PFS in comparison to docetaxel alone and also increased the toxicity. [202] The results of the phase I study evaluating the combination of resminostat and S-1 (a fluoropyrimidine used as second-line treatment for BTC therapy) revealed that with resminostat (5 days on/2 days off, second dosage regimen for the combination therapy) was well tolerated. [201]
Pracinostat (SB939)	Oral HDAC inhibitor Pracinostat demonstrated clinical benefits and notable activity in phase II study conducted in patients with intermediate or high risk MF [203] In another phase II study, pracinostat was found to be well tolerated in patients with advanced solid tumours. The outcome of the investigation recommended the dose of 60 mg on a schedule of 5 consecutive days every 2 weeks. [204] In another study (Phase II), pracinostat was well tolerated in children with refractory solid tumors [205] Pracnitostat is currently undergoing the below mentioned studies: Newly diagnosed AML (Pracinostat in combination with azacitidine) (Phase 3, NCT03151408) High risk MDS (Pracinostat and Azacitidine, NCT03151304, status—unknown, Phase 2)
Givinostat	Developed by Italfarmaco Orally bioavailable hydroxamate inhibitor of HDAC [206–208] A phase 3 clinical stage evaluation of safety and efficacy of givinostat in comparison to hydroxyurea in JAK2V617F + high-risk PV Patients is planned to start in 2021. [208] In a phase 2 clinical stage evaluation, givinostat was assessed for safety and efficacy in patients with JAK2^V617F^ positive myeloproliferative neoplasms. CR and PR were attained in the study indicating that givinostat holds enough promise for further clinical exploration in patients with MPN [206] A phase 2 study evaluating the efficacy of givinostat in JAK2V617F + patients with PV was conducted. The study results led to the determination of MTD as 100 mg twice daily. Overall response rate was found to be 80.6%. Normalization of haematological parameters was seen in majority of the patients. Grade 1/2 thrombocytopenia and gastrointenstinal disorders were observed as the common adverse effects. Overall, givinostat was well tolerated. 11% complete and 89% partial response rates were observed as the long term results of the phase II clinical trial. To add on, a lower incidence of thrombotic events in comparison to historical controls treated with hydroxyurea along with good tolerability were observed in JAK2V617F + PV patients. [NCT00928707] [207, 208]
Abexinostat	Developed by Xynomics A pan HDAC inhibitor Significantly durable responses were demonstrated by a combination of pazopanib and abexinostat in patients with clear cell renal cell carcinoma. An ongoing response of > 5 years duration was observed in one patient with previous refractory disease. In peripheral blood mononuclear cells, histone acetylation induction was associated with durable treatment response. [209] Abexinostat is undergoing phase 3 clinical investigation for advanced and metastatic renal cell carcinoma. (NCT03592472, status—active, not recruiting) Assessment study of Abexinostat for safety and efficacy in Patients with R/R FL was initiated on April 22^nd^, 2020 On 23^rd^ September, Abexinostat was granted a fast track designation from the US FDA as 4L therapy treating FL. Earlier, abexinostat in combination with pazopanib, as a first- or second-line treatment of renal cell carcinoma received fast track designation from FDA. [210]
AR-42	An oral pan –HDAC inhibitor In a phase I dose escalation clinical trial, AR-42 was found to be well tolerated with no DLTs. 40 mg (three times weekly for three weeks of a 28-day cycle) was determined as the MTD. Disease control demonstrated by one patient each with MM and mantle cell lymphoma for 19 and 27 months respectively. Reduction of serum CD44 was observed in the treatment. Overall, AR-42 was found to be safe (NCT01129193) [211] In a phase 1 study, AR-42 was administered (3 dose levels (DL): AR-42 20 mg qd on d1,3,5 in DL1, 40 mg qd on d1,3,5 in DL2 and 40 mg qd on d1,3,4,5 in DL3) to thirteen patients with previously untreated or R/R AML. Decitabine was administered to the patients at the dose of 20 mg/m^2^ on day 6–15 of each induction cycle and 20 mg/m^2^ on day 6–10 of each maintenance cycle. The results of the study indicated a DLT of polymicrobial sepsis. At DL3, multi-organ failure occurred. CR was observed in two patients. CR for an ORR of 23.1% was observed in one patient. The biologic endpoint was not met in this study. [212]
Bisthianostat	A novel orally available bisthiazole-based HDAC inhibitor Comprises of thiazole-thiazoline as the capping unit in natural product largazole [213] A phase 1 study conducted in patients (8 patients 8 patients enrolled at 3 dose levels) from 100 to 400 mg with R/R multiple myeloma. In the study, hematological TAAEs were observed in 4 of 8 patients (50%). Grade 3/4 hematological as well as non-haematological AEs were not observed. There was no discontinuation in the treatment of patients due to AEs except one of the patient who experienced grade 2 nausea. Overall, the outcome of the investigation indicates that the drug is well tolerated and is endowed with modest efficacy. Stable disease (SD) was evidenced 50% patients. [214] (NCT03618602)
Quisinostat	Orally available potent HDAC inhibitor [215–217] In a phase II study, the combination of quisinostat with paclitaxel and carboplatin in subjects with recurrent platinum resistant ovarian cancer demonstrated high efficacy and good tolerability. [NCT02948075] [216] Quisinostat was evaluated in patients with previously treated CTCL in a phase II multicentre trial. In the study, quisinostat (8 mg or 12 mg on days 1, 3 and 5 of each week in 21-day treatment cycles) was administered to the patients. The results of the study demonstrated that quisinostat 12 mg three times weekly was found to be effective and safe for the treatment of patients with R/R CTCL [217] A phase 1 study involving the administration of quisinostat (orally, once daily in three weekly cycles) to patients with advanced malignancies demonstrated better toleration of intermittent schedules than the continuous schedules. Overall, quisinostat displayed an adverse event profile similar to other HDAC inhibitor and the RP2D was determined to be 12 mg [215] (NCT00677105)
Nanatinostat	An oral HDAC inhibitor selective for specific isoforms of Class I HDACs Induces latent viral genes in EBV-associated malignancies A combination of nantinostat with antiviral valganciclovir for the treatment of EBV-associated R/R lymphomas was evaluated in a Phase 1b/2a study. The results of the study are mentioned below: ORR—56% (Phase 1b portion), Complete response—28%, clinical benefit rate—78%, Median duration of treatment for responders—6.5 months In HIV-negative patients, ORR—67%, CR—33% and CBR—93% Overall, the combination was well tolerated at 5 mg (Nanatinostat) and 450 mg (valganciclovir) BID. Hematological adverse events were observed that were resolved without sequalae or bleeding events. [218], NCT03397706] Fast Track designation has been granted to the combination of nantinostat and valganciclovir by FDA for the treatment of patients with R/R EBV-positive lymphomas. [219]
Fimepinostat	A synthetic, orally-available, small molecule inhibitor of HDAC and PI3K In a phase 1 clinical study of fimepinostat conducted in patients with R/R/ lymphomas or multiple myeloma, OR was observed in 9 patients in 21 response-evaluable patients out of 25 patients with R/R DLBCL enrolled in the study. 60 mg dose of fimepinostat (administered orally) using a 5 days on and 2 days off schedule in a 21-day cycle was determined as the RP2D. At the RP2D, no DLTs were observed Orphan drug designation was granted to Fimepinostat by FDA for the treatment of patients with DLBCL in 2015 In 2018, Fast track designation was granted to by US FDA for the development of fimepinostat in adult patients with R/R DLBCL. [220]
Etinostat, (SNDX-275 and MS-275) [222]	Developed by—Syndax Is an orally administered selective class I HDAC inhibitor Belongs to the benzamide class of HDAC inhibitors A combination study (Phase 3) of Exemestane and Entinostat in Chinese subjects with Hormone Receptor-Positive, Locally Advanced or Metastatic Breast Cancer is also ongoing (NCT03538171) The effects of entinostat addition to exemestane in patients with HR-positive advanced breast cancer were evaluated. The results of this phase II clinical study (ENCORE 301) revealed remarkable advantages attained with this combination in terms of improvement of OS as well as PFS. In light of these favourable trends, a phase III trial (E2112) was initiated for the evaluation of this combination in patients with locally advanced and metastatic breast cancer (NCT02115282). [222] The combination studies of etinostat with pembrolizumab (ENCORE601, phase 2 trial) conducted in subjects with NSCLC demonstrated better outcomes attained with this combination in patients with higher levels of a circulating cell called a classical monocyte [223] An assessment study of High Dose Interleukin 2 vs High Dose Interleukin 2 in combination with Entinostat in Advanced Renal Cell Carcinoma is ongoing. (NCT03501381, Recruiting)
Mocetinostat,	Recently, first clinical trial for mocetinostat using genomic-based selection to identify patients with urothelial cancer was conducted. The results of the study demonstrated that administration of mocetinostat led to significant toxicities and displayed limited efficacy. Owing to this, mocetinostat monotherapy is not recommended for further investigation in this setting [224] Mocenitostat was evaluated in a phase 2 study in patients (n = 72 with R/R DLBCL and FL). The study design involved the administration of mocetinostat (starting doses: 70–110 mg TIW, 4-week cycles). The results of the study demonstrated that 54.1% and 73.1% of patients showed clinical benefit (response or stable disease) from mocetinostat in the DLBCL and FL cohorts and PFS was 1.8–2.8 months (DLBCL) and 11.8–26.3 (FL). Fatigue, nausea and diarrhoea (61.1%) were found to most frequent TAAEs. Overall, mocenitostat was found to be safe as single agent, however, was endowed with limited efficacy. [225] Mocetinostat is undergoing the below mentioned studies: Metastatic Solid Tumors and NSCLC (combination with PD-L1 Inhibitor, Durvaluma, Phase 2, NCT02805660, status—completed, results not posted) Advanced NSCLC (combination with nivolumab, NCT02954991, Phase 2, status—recruiting) In children, Adolescents and young adults with Refractory and/or Recurrent Rhabdomyosarcoma (NCT04299113, status—recruiting, Phase 1) Advanced NSCLC (combination with pembrolizumab plus guadecitabine, Phase 1, NCT03220477 status—recruiting)
Tucidinostat (Chidamide)	Developed by chipscreen Is an orally bioavailable HDAC inhibitor belongs to benzamide class of HDAC and inhibits HDAC isoenzymes 1, 2, 3 and 10 CFDA approved (for PTCL) [226] Currently, tucidinostat is undergoing phase III clinical evaluation in combination with R-CHOP in patients with newly diagnosed MYC/BCL2 double-expressor DLBCL (NCT04231448, status—recruiting) In a Phase III study, combination of exemestane with tucidinostat for post-menopausal patients with advanced, hormone receptor-positive breast cancer was assessed for safety and efficacy. In the study, 365 patients were enrolled (244 tucidinostat and 121- placebo). In the tucidinostat group, the median duration of follow-up was 13.9 months (IQR 9·8–17·5) and investigator-assessed median PFS was 7·4 months (95% CI 5.5–9.2). Overall, the combination improved the PFS in comparison to placebo plus exemestane. Haematological adverse events (Grade 3–4) were more common with the combination than the placebo plus exemestane group [227] Tucidinostat in combination with R-CHOP is also undergoing phase III evaluation in patients with newly diagnosed MYC/BCL2 Double-Expressor DLBCL (NCT04231448) and phase II evaluation in combination with toripalimab in subjects with refractory and advanced soft-tissue sarcoma (NCT04025931, Phase, recruiting) The combination of tucidinostat with exemestane has been approved by NMPA for breast cancer [235]
Domatinostat	Is an orally administered, selective inhibitor of LSD1 and class 1 HDAC inhibitor In a phase 2 studies of domatinostat in patients with patients with advanced hematological malignancies, domatinostat (monotherapy) demonstrated clinical activity and was found to be safe, well tolerated and the RP2D was determined to be 400 mg TDD in a 200 mg BID schedule (14 + 7) [228] Domantinostat is currently undergoing phase 2 evaluation (combination studies with avelumab) in GI cancer (NCT03812796)
CXD1	Developed by celleron therapeutics CXD101, is a dual mechanism HDAC inhibitor Is a novel epigenetic immune-regulator endowed with potential to enhance immune recognition of tumour cells CXD-101 has been found to be effective in lung and colon xenograft models in preclinical studies CXD-101 is undergoing phase 2 clinical studies in NSCLC (NCT03833440, status—recruiting) Clinically important tumour remissions have been displayed by CXD101 in the phase I trials. [229]

The results presented in Table 3 clearly indicate that HDAC inhibitors have demonstrated promise in oncology indications as single agents. Selective toxicity in liver cancer exerted by tefinostat [236], encouraging results of clinical trials evaluating ricolinostat [230] in multiple myeloma, motivating results attained with aromatase inhibitor, exemestane and the *ortho*aminoanilide entinostat in the treatment of postmenopausal breast cancer [237], combination of exemestane and tucidinostat receiving NMPA approval for the treatment of breast cancer [227], advancement of the combination of pracinostat and azacytidine to higher stage clinical investigation for the treatment of MDS and newly diagnosed AML [238, 239] exemplify some of the successful clinical stage investigations trials conducted in the recent past.

### LSD1 inhibitors

**Fig. 6:**
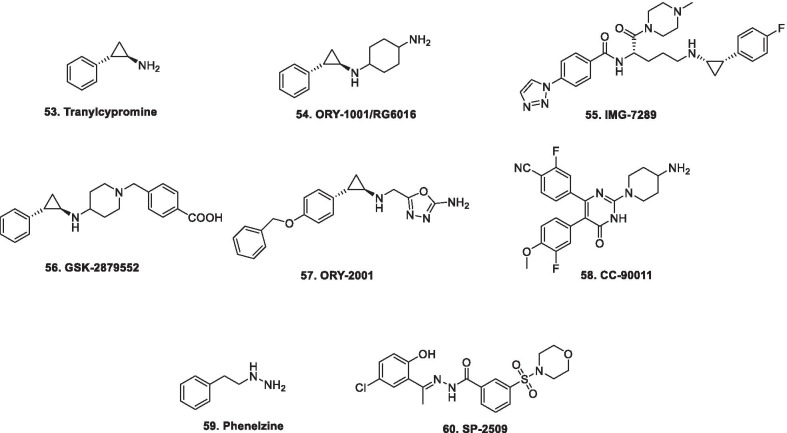
LSD1 inhibitors

Histone demethylase LSD 1 is reported to be overexpressed in diverse malignancies. Growth inhibition of multiple tumor types is exerted via inactivation or knockdown of LSD1 in cancer cells [240–243]. Literature survey indicates that only those inhibitors that specifically targets lysine specific histone demethylase 1A (KDM1A) have been able to advance to clinical stage investigation [244, 245]. In light of the aforementioned, inhibition of LSD1/KDM1A is presently being given serious consideration for the fabrication of new antitumor scaffolds. The pipeline of LSD1 inhibitors is filled with numerous candidates undergoing clinical stage investigation in monotherapy as well as combination therapy namely tranylcypromine, phenelizine sulfate, ORY-1001, GSK-2879552, IMG-7289, INCB059872, CC-90011, and ORY-2001 (Fig. 6). This section will present an overview of the results of clinical trials of the LSD1 inhibitors (Table 4).

**Table 4:** Update on LSD1 inhibitors

LSD1/ KDM1A inhibitor	Details	Clinical trials
Tranylcypromine	A monoamine oxidase inhibitor used in clinic for the treatment of depression It is as an irreversible and weak LSD1 inhibitor It exerts irreversible inhibition of LSD1 through the formation of a covalent adduct with FAD cofactor of LSD1 [246–248] Tranylcypromine restores sensitivity to all-trans retinoic acid in AML [249]	A study to determine the RP2D of tranylcypromine in combination with fixed-dose ATRA and AraC (cytarabine) was initiated on March 24, 2016. (Phase I/II) The details of the clinical study are as follows: N = 60 (Goal), currently enrolling Treatment—TCP (20,40,60,80 mg) + ATRA g/m^2^ + AraC 40 mg on days 1–10 Study results—not yet posted (NCT02717884) Safety and efficacy of the combination of tranylcypromine (TCP) and all-trans retinoic acid (ATRA) was evaluated in a phase 1 study in patients with R/R AML and high-grade MDS [250]. The details of the clinical study are as follows: N = 15 (8 AML, 7 MDS) Treatment—ATRA 45 mg/m^2^ TCP 20 mg/m^2^ OI BID (MTD) TAAEs—Grade 1 and 2 (majority) Grade 3/4 AE (> 10%) includes fever, thrombocytopenia, anemia and lung infection. Best evaluable responses included 5 patients with prolonged SD > 3 (2 AML, 1 CMML, 2 MDS), 1 marrow CR (MDS) and 1 MLFS (AML) Overall the combination was found to be endowed with clinical activity and demonstrated an acceptable safety profile [250] (NCT02261779) Another study (open-label, dose escalation, phase 1 study) to evaluate the efficacy of the combination of TCPA and all-trans-retinoic acid in patients with AML and MDS was also initiated on October 23, 2014. (NCT02273102) Status—active, not recruiting
ORY-1001	Developed by Oryzon Genomics ORY-1001, a tranylcypromine derivative, binds covalently to FAD and is a potent, selective and irreversible inhibitor of LSD-1 (IC_50_ = 18 nM) [251] ORY-1001 was found to induce time and dose dependent accumulation of H3K4me2 at LSD1 target genes It induced cell apoptosis of THP-1 cells and inhibits colony formation and cell proliferation of MV(4;11) (MLL-AF4) cells (EC50 < 1 nM) Oral administration of ORY1001 (< 0.020 mg/kg daily) caused substantial reduction of tumor growth in MV (4;11) xenografts after oral administration of < 0.020 mg/kg daily ORY-1001 is stable in hepatocytes, possesses excellent oral bioavailability, activity, and target exposure in vivo [252] Synergistic efficacy attained against human AML blast progenitor cells (BPCs) was recently demonstrated via co-treatment with ORY-1001 and BET protein inhibitor OTX015 [253] ORY-1001 exhibits synergism with ATRA, cytosine arabinoside, and quizartinib), selective epigenetic and targeted inhibitors (e.g., EPZ5676, SGC-0946, decitabine, azacitidine, SAHA, and ABT-737) in the in vitro studies (MV (4;11), MOLM13, and MOLT4 cell lines) and also led to growth suppression of AML xenograft model. [254, 255]	ORY-1001 was evaluated in a phase 1 study in patients with acute leukemia (EUDRACT 2013-002447-29) and the results demonstrated that ORY-1001 was well tolerated at the recommended dose and promoted the differentiation of blast cells (64% patients). [252]. The details of the study are mentioned below: N = 27 (26 AML, 1 ALL) Treatment: ORY-1001 140mcg/m2/day 5 days week for 28-day cycle DLTs: TCP, lobar pneumonia, febrile neutropenia (in combination with grade 2 fatigue and grade 2 erythema multiforme) Efficacy: OR in 5 (35%, 5 of 14), PR in 3 and SD in 2 patients. 9/14 patients had blast differentiation Overall the results of the phase I clinical investigation of ORY-1001 in AML demonstrates the potential of the LSD1 inhibitor to exert therapeutic benefit. Some hematological toxicities such as thrombocytopenia and neutropenia were observed. [255] A phase 1 study of ORY-1001 (orally administered) in patients with relapsed, extensive stage disease SCLC (NCT02913443) has been completed but the results have not been presented in public domain
GSK2879552 (GSK)	An irreversible tranylcypromine based LSD1 inhibitor Endowed with activity in AML and SCLC GSK2879552 decreased the cell proliferation in 19 of 25 AML cell lines and decreased blast count formation in 4 of 5 primary AML bone marrow samples A notable reduction in GFP + cells and prolonged OS in comparison to control treated mice was evidenced in mice transplanted with AML cells. Moreover, surface expression of CD11b and CD86 was also observed [256]	Clinical evaluation of GSK2879552 (Phase 1//II) as a single agent as well as in combination with azacitidine in patients with MDS was also terminated (NCT02929498) A clinical investigation (Phase 1 dose escalation study) of a combination of GSK2879552 and ATRA in patients with R/R AML was terminated (NCT02177812) Another Phase 1 trial to evaluate the safety, efficacy, pharmacokinetics and pharmacodynamics in patients with SCLC was also terminated (NCT02034123)
INCB059872	Developed by Imago BioSciences It is a tranylcypromine based LSD1 inhibitor endowed with remarkable antiproliferative effects towards AML, Ewing’s sarcoma, SCLC, and prostate cancer (preclinical tumor models) [257–260] Reduced proliferation and induced differentiation in AML cell lines and human AML cells was evidenced with INCB059872 (in vitro treatment) [259] INCB05982 induced differentiation of blasts, reduced blast count, normalized counts, and prolonged survival consistent with anti-leukemic effects in the MLL-AF9 AML mouse model. [259] Revelations from a preclinical investigation indicates synergistic effects of INCB059872 with ATRA in non-APL AML l systems [261]	Evaluation of INCB-59872 to assess safety, tolerability and antitumor activity (open label phase 1b study) is currently recruiting participants with R/R Ewing sarcoma (NCT03514407 and EudraCT 2018–000,062-11) A dose escalation/dose expansion study of INCB059872 aimed at establishing the safety as well as tolerability profile of INCB059872 in subjects with advanced malignancies (AML/MDS, SCLC, MF, Ewing sarcoma, and poorly differentiated neuroendocrine tumors) was started in May, 2016 (NCT02712905, status—recruiting) A phase 1/2 study to evaluate the safety and tolerability of INCB059872 in combination with pembrolizumab and epacadostat in subjects with advanced or metastatic solid tumors (NCT02959437) was carried out. The results of the study have not been disclosed Phase I/II ABNL-MARRO trial for MDS or myeloproliferative disorders (NCT04061421, status—Not yet recruiting A safety and biological activity evaluation study of INCB059872 in patients with sickle cell disease was recently terminated on March 1,2019 and the reasons stated for the termination were business oriented (NCT03132324)
IMG-7289 (IMG)	Developed by Imago BioSciences Tranylcypromine based irreversible lsd1 inhibitor IMG-7289 has demonstrated potential to inhibit the inflammatory cytokines production In a study, treatment with IMG-7289 led to impairment of self-renewal and proliferation of neoplastic stem cells and also exhibited disease-modifying ability in MF Studies conducted in mouse models of myeloproliferative neoplasms indicates that IMG-7289 decreased the elevated cell counts, inflammatory cytokines, mutant allele frequencies, spleen size, marrow fibrosis. [243, 262]	The IND application of IMG-7289 was accepted in 2018 by FDA for conducting the clinical investigation in MF [263] IMG-7289 is currently being evaluated in the below mentioned studies: Phase 2b study of IMG-7289 in patients with MF, status – currently recruiting (NCT03136185) A Phase 2 investigation of IMG-7289 (oral administration, once daily) patients with essential thrombocythemia (NCT04081220), status – currently recruiting Clinical evaluation of IMG-7289 with or without ATRA in patients with AML and MDS is completed (Phase 1, NCT02842827). The results are as follows: N = 45, IMG-7289 ± ATRA 45 mg/m2 RP2D of IMG-7289 = 0.25 mg/kg Overall, IMG-7289 was well tolerated in the study
CC-90011	Developed by Celgene Is the first reversible LSD1 inhibitor in clinical trials that has demonstrated efficacy in advanced solid tumors and R/R NHL, particularly in patients with neuroendocrine tumors [264, 265]	A study to evaluate the safety and efficacy of CC-90011 in patients with R/R Solid tumors and Non-Hodgkin's lymphomas was initiated on August 23^rd^, 2016, status – recruiting A phase 2 study was recently initiated to test the safety, tolerability, and preliminary efficacy of CC-90011 in combination with approved anticancer in patients with advanced stage SCLC (NCT03850067), status – recruiting
Phenelzine	A non-selective and irreversible monoamine oxidase inhibitor, Phenelzine, belongs to the hydrazine class and is used in clinic as an antidepressant and anxiolytic It is LSD1 mechanism based inactivator similar to tranylcypromine, however utilizes the hydrazine functionality to initiate the key steps (mechanism) unlike the cyclopropyl oxidation (evidenced with Tranylcypromine) [266]	Phenelzine sulfate is undergoing clinical evaluation with Abraxane (Protein bound chemotherapy—combines paclitaxel with albumin) (NCT03505528)
SP-2509 (SP)	It is a reversible, selective and allosteric inhibitor of LSD1 (IC_50_ = 13 nM) It is not active against MAO-A and B [267, 268] The outcome of a study has revealed that SP-2509 could induce differentiation of AML cell lines and primary AML. In addition, overall survival was prolonged in AML mouse models [267]	A clinical analog of SP-2509, SP-2577 is currently undergoing phase 1 clinical investigations in R/R Ewing’s sarcoma (NCT03600649)

### BET inhibitors

**Fig. 7:**
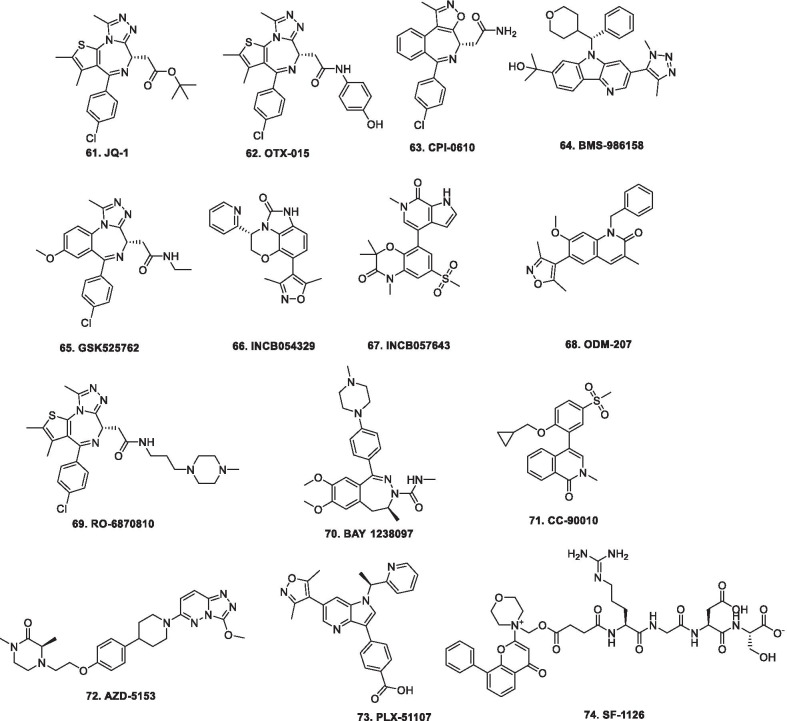
BET inhibitors

The bromodomain and extra-terminal domain (BET) family of bromodomain containing proteins are epigenetic readers that are considered to be important regulators of the epigenome owing to their ability to recognize N-acetyl lysine (KAc) post translational modifications on histone tails [269]. Among the bromodomain containing proteins, BET family (BRD2, BRD3, BRD4, and BRDT) has been the most extensively explored and the results of these explorations has ascertained its links with diverse cancers. In light of the aforementioned, the BET family of proteins represents a well-established therapeutic target for oncology and immunoinflammation indications [270–272] and numerous small molecule inhibitors capable of abrogating the BET − KAc interactions are currently under clinical investigation (Fig. 7). This section will present an update on BET inhibitors undergoing clinical studies.

*JQ1* is a thienotriazolodiazepine that represents the first generation synthetic BET inhibitor. It potently inhibits BRD2, BRD3, BRD4, and the testis-specific protein BRDT in mammals. JQ-1 is endowed with anticancer efficacy in murine and xenograft models of NMC, AML, multiple myeloma, and Burkitt's lymphoma. The preclinical study results have reported toxicities associated with the use of JQ1 that included intestinal crypts disruption in mice with reduced BRD4 levels, impaired long-term memory and heightened anxiety, depletion of hematopoietic cells, skin hyperplasia, and; neuronal defects and obesity in mice with reduced BRD2 levels. Though JQ1 is not being evaluated at the clinical level, several structurally related BET inhibitors are being clinically evaluated for diverse malignancies [273].

*OTX-015* is a thienotriazolodiazepine BET inhibitor that selectively blocks BRD2/3/4 and is the first BET inhibitor to be evaluated clinically [274]. It is endowed with striking antitumor activity against a panel of cell lines derived from hematologic malignancies and solid tumors (breast and prostate cancer, neuroblastoma and glioblastoma [275–281]. OTX015 was administered orally in a dose-escalation, phase Ib study in patients with haematologic malignancies. In the study, analysis of the blood samples from 81 patients was performed that were administered OTX015 (dose: 10–160 mg or 40 mg twice daily). The results of the study demonstrated that OTX015 pharmacokinetics can be adequately described by a one compartment open model with linear elimination. The absorption rate constant (ka) = 0.731 h^-1^, V = 71.4 L and CL = 8.47 L h^-1^ were the estimated pharmacokinetic parameters. Overall, the results from population pharmacokinetic modelling of OTX015 plasma concentrations indicated that there is need for dose adjustment [282] (NCT01713582). In a dose escalation study conducted in patients with acute leukaemia, OTX015 was administered orally at doses increasing from 10 mg/day to 160 mg/day (14 of 21 days). The results of the study recommended the administration of 80 mg OTX-015 (once daily) on a 14 days on, 7 days off schedule [283, 284] (NCT01713582). OTX-015 demonstrated a dose-proportional exposure and a favorable tolerance profile in solid tumor patients along with reasonable activity in NUT midline carcinoma patients (Phase 1b clinical trial). In addition, clinical improvement was evidenced in heavily pre-treated castrate-resistant prostate cancer patients. The RP2D was deduced to be 80 mg QD [oral OTX-015 continuously (21/21)] [285] (NCT02259114). A Phase 1 study of OTX-015/MK-8628 in patients with advanced solid tumors (NCT02698176) and a phase 2 study of OTX-015/MK-8628 in GBM patients were terminated owing to lack of clinical activity/ limited efficacy and some safety related concerns (NCT02296476). Another phase 1 clinical study of OTX015/MK-8628 in patients with selected hematologic malignancies (MK-8628-005) is currently active (not recruiting) (NCT02698189).

*CP-0610 *CP-0610 is a potent and selective benzoisoxazoloazepine BET bromodomain inhibitor. A preclinical study results demonstrated that CP-0610 attenuated BET-dependent gene expression in vivo and was found to be endowed with antitumor efficacy in an MV-4-11 mouse xenograft model [286]. A first-in-human Phase 1 study of CPI-0610, was conducted in subjects with R/R lymphoma. The study design involved oral administration of CPI-0610 once daily (QD) on days 1–14 of a 21-day cycle. The number of patients enrolled were 44. The results of the study demonstrated that CPI-0610 was well tolerated. However, reversible and non-cumulative dose dependent thrombocytopenia was the principal toxicity evidenced. Though the study results could not determine MTD, clinical efficacy was observed [287]. A phase 1 study (study design: 3 + 3) of CPI-0610 (dose = 6–300 mg) in capsule form and at 125 mg or 225 mg in tablet form in patients (n = 64) with progressive lymphoma was conducted. Determination of MTD was the primary end point of the study which was deduced to be 225 mg in tablet form. Thrombocytopenia (42.2%), nausea (17.2%), and fatigue (17.2%) were the common TAAEs. The efficacy analysis results conducted in 38 patients demonstrated 5 OR (13.2%), including 2 CR and 3 PR. (recruitment status: complete, NCT01949883) [288]. CPI-0610 was also evaluated clinically in a phase 1 study (study type interventional) in subjects (30 participants) with previously treated multiple myeloma. The study was initiated on July, 2014 and completed on November 2017 (study results—not posted yet, NCT02157636). A phase 2 clinical investigation of CP-0610 in patients with malignant peripheral nerve sheath tumors was recently withdrawn due to lack of enrolment (NCT02986919). In light of the significant clinical activity evidenced preclinically and clinically (Phase 1 studies), CP1-0610 is currently being evaluated in monotherapy and in combination with ruxolitinib in patients with MF (NCT02158858). Evaluation of the spleen volume response and effects on transfusion independence rate are the primary objectives of the study. [289].

*BMS-986158* a BET bromodomain inhibitor that downregulates c-Myc expression and causes cancer cell death in c-Myc-driven cancer cell lines in vitro. It is endowed with inhibitory potential towards tumor growth in triple-negative breast, colorectal, and lung cancer patient-derived mouse xenograft models [290]. In a phase I/IIa dose escalation study (advanced cancer), BMS-986158 (0.75, 1.25, 2.0, 3.0, or 4.5 mg) was administered to patients (n = 69, once daily). BMS-986158 displayed a Tmax of 2–4 h, T1/2 of 33–82 h, and linear PK. Reversible thrombocytopenia was observed as the DLT. Adverse events were observed in 63% patients (diarrhea—34%, thromobocytopenia, 28%, fatigue 16%). Out of the four patients with NMC, (dose: 2 mg, Schedule A, 279 days), one patient experienced a 16% tumor reduction. CCR2 and HEXIM1 were the biomarkers examined [291]. BMS-986158 is currently undergoing evaluation in pediatric cancer (NCT03936465, status recruiting) and a phase 1/2 clinical evaluation to assess safety, tolerability, pharmacokinetics, and pharmacodynamics of BMS-986158 (advanced cancers) (NCT02419417, status recruiting).

#### ZEN003694

It is an orally available small molecule selective BET bromodomain inhibitor ZEN-3694 that demonstrates > 20-fold selectivity over non-BET bromodomains, thereby causing an inhibition of interaction of acetylated histone peptide at IC_50_ values in low nanomolar range). ZEN-3694 displays synergistic efficacy with many standard of care and targeted therapies in diverse malignancies and is particularly effective against CRPC and TNBC xenograft models [292]. The safety and tolerability of ZEN003694 (phase 1 study) in patients (n = 44) with mCRPC (NCT02705469) was completed on October 2017 (results not posted yet).

The combination studies of ZEN-3694 and enzalutamide in patients with mCRPC was conducted. The study design involved a 3 plus 3 dose escalation scheme (starting dose: 36 mg ZEN-3694 + ENZ 160 mg, daily oral dose). Doses were expanded in parallel cohorts (ZEN-3694 −48 and 96 mg daily) and 64 patients were enrolled in the study. Overall, the outcome of the study was quite optimistic and demonstrated that ZEN-3694 is endowed with an acceptable safety and PK profile. The combination could also attain promising disease stabilization warranting its further investigation. The study results are summarized as: adverse events—transient photophobia (66%), nausea (40%), fatigue (31%), decreased appetite (22%), and dysgeusia (16%). No Grade ≥ 3 thrombocytopenia was observed, overall median TTP was determined to be 44.4 weeks (NCT02711956) [293]. A phase 2 study of ZEN-3694 in combination with talazoparib in patients with TNBC without germline mutations of BRCA1 or BRCA2 is currently recruiting (NCT03901469). The clinical evaluation of ZEN-3694 in combination with enzalutamide plus pembrolizumab in patients with mCRPC is expected to start on August 3^rd^, 2020 (Current status: not yet recruiting). The study design is as follows: clinical stage: Phase 2, Study type: open lable, non randomized Interventional, Enrollment (estimated): 54.

#### Molibresib (GSK525762)

Endowed with antiproliferative effects evidenced in preclinical studies models of NC and other solid and hematologic malignancies, GSK25762 is an orally bioavailable, small-molecule BET inhibitor [294]. The phase 1 study of GSK525762 was conducted in patients with nuclear protein in testis (NUT) carcinoma (NC) and other solid tumours to determine the RP2D and establish the pharmacokinetic as well pharmacodynamics profile. The study design involved administration of GSK525762 (orally once daily, 3 + 3 design, dose escalation study, n = 65) starting with a dose of 2 mg/d. Grade 4 thrombocytopenia encountered with molibresib 60–100 mg along with gastrointenstinal events were the most frequent TAAEs. 80 mg once daily was determined as RP2D. GSK52576 exhibited rapid absorption and elimination (maximum plasma concentration: 2 h; t1/2: 3–7 h). Reductions in circulating monocyte in circulating monocyte chemoattractant protein-1 levels were also observed. Four patients out of 19 patients attained confirmed or unconfirmed PR, eight had SD stable and four were progression-free (> 6 months) [295] (NCT01587703). Clinical activity was also evidenced in a phase 1 trial conducted in patients (n = 46) with AML. The results of the study are as follows: PRs—3, CRs—2, AEs—dysgeusia (37%), diarrhea (33%), and nausea (28%). Overall the drug was found to be safe and effective. [296] An open label study in patients with NHL was conducted and the RP2D was determined as 60 mg QD. Response rate (50%) was observed in patients with CTCL. Manageable thrombocytopenia was observed that was monitorable and reversible. [297]. Evaluation (pharmacokinetics, pharmacodynamics and clinical activity) of GSK525762 (dose escalation study) for R/R hematologic malignancies has recently been completed, however, the results have not been posted yet. (NCT01943851). GSK525762 is currently being evaluated in combination with fulvestrant in patients with HR + / HER2− advanced or metastatic breast cancer. The expected completion date of the study is September 28th, 2020 (NCT02964507, status: active, not recruiting). GSK525762 is also currently being evaluated in combination with androgen deprivation therapy and other agents in patients with castrate-resistant prostate cancer (NCT03150056, status- active, not recruiting).

#### GSK2820151

A potent and selective small molecule BET inhibitor, GSK2820151, has demonstrated inhibitory potential towards the proliferation of several solid tumour cell types along with in-vivo activity in xenograft models [298]. A phase 1 clinical investigation of GSK2820151 in patients with advanced or recurrent solid tumours was recently terminated to focus on the clinical growth of another BET inhibitor, GSK525762, owing to established risk benefit profile. (NCT02630251).

#### INCB054329

A structurally distinct BET inhibitor that has demonstrated potency against B-cell malignancies in preclinical models. A phase 1/2 study of INCB054329 in patients with advanced malignancies was terminated due to pharmacokinetic issues (NCT02431260).

#### INCB057643

INCB057643 is a potent and selective small-molecule BET inhibitor [299]. Phase I/II clinical trial was conducted in patients with solid tumor (NCT02711137) and the study involved the oral (continuous) administration of INCB057643 (once daily) in 21 days cycles (3 + 3 design). The results of the study are as follows: TAEs—increased conjugated bilirubin, increased INR, TCP, decreased appetite and dyspnea, 1 patient with SD ≥ 6 months (11 of 13 patients evaluable for efficacy in part 1). 2 patients (evaluable for efficacy, Part 2) of 14 patients with solid tumors had PD. Overall, the outcome of the study demonstrated clinical activity of INCB057643. The study is now terminated owing to safety issues [NCT02711137)] [300]. Clinical investigation of INCB057643 in patients with MF started on August 31, 2020. (NCT04279847). A phase 1/2 trial was started on January 26 2017 to evaluate the efficacy of pembrolizumab, epacadostat with INCB057643 (NCT02959437).

#### ODM-207

ODM-207 is a novel, potent and highly selective BET bromodomain inhibitor. In preclinical studies, it has demonstrated significant efficacy against prostate and breast cancer. [301]. ODM-207 was evaluated for safety and pharmacokinetics in patients with selected advanced solid tumours (Phase 1/2 study) (NCT03035591). The study has been completed but the results have not been published yet.

#### RO6870810

RO6870810 is a small-molecule BET inhibitor. A clinical stage investigation (Phase 1, two parts) was conducted to evaluate the safety, PK, tolerability, and efficacy in subjects (n = 84, 54 in part A and 30 in part B) with advanced solid tumors. The study was completed on October 2017, but results have not yet been published (NCT01987362). Another phase 1 study of RO6870810 in subjects with AML and MDS was completed on August 2017 but the results have not been posted. (NCT02308761). The phase 1 studies of RO6870810 (mono and combination therapy in advanced multiple myeloma was also recently completed. (NCT03068351). A combination study of RO6870810 and Atezolizumab (PD-L1 Antibody) in participants with advanced ovarian cancer or TNBC was terminated (NCT03292172). A combination study (phase 1) of RO6870810 and venetoclax, with or without Rituximab, in patients with DLBCL was recently completed but the results have not been posted. (NCT03255096).

#### BAY 1238097

BAY 1238097 is a highly selective and potent BET inhibitor. In a preclinical study, BAY 1238097 demonstrated tumour growth inhibitory potential in xenograft mouse models of lymphoma [35]. A phase 1 study of BAY1238097 was conducted in patients with cytologically or histologically confirmed advanced refractory malignancies. BAY 1238097 was administered orally (twice weekly in 21-day cycles) employing a dose escalation strategy with a starting dose of 10 mg/week. The results of the study demonstrated prolonged SD in two patients (no responses observed). Increased HEXIM1 expression and decreased *MYC* expression was also observed. The study was terminated owing to the occurrence of DLTs at dose below targeted drug exposure. (NCT02369029) [302].

#### CC-90010

It is an oral and reversible BET inhibitor endowed with significant activity in lymphoma and solid tumor cell lines [303]. CC-90010 was evaluated in subjects with advanced solid tumors and R/R NHL (R/R NHL). In the study, 69 patients were enrolled (67 with solid tumors and 2 with R/R NHL). In 17 patients, grade 3/4 TAEs were observed that included (≥ 2 pts) thrombocytopenia (7%), platelet count decreased (4%), fatigue (3%), and increased alanine aminotransferase (3%). PR was observed in 2 patients; prolonged SD was observed in 7 patients (SD > 9 months). Overall, preliminary clinical activity was observed in the study with manageable TAAEs. (NCT03220347) [303]. The phase 1 clinical evaluation of CC-90010 in progressive/recurrent diffuse astrocytoma, anaplastic astrocytoma and glioblastoma started on August 2, 2019 (Current status, NCT04047303). Phase 1 combination studies of CC-90010 with temozolomide (with or without radiation therapy) in patients with newly diagnosed glioblastoma is underway (current status—not yet recruiting, NCT04324840). A phase 1/2 investigation of CC-90011 in combination with cisplatin and etoposide in patients with SCLC is also underway (status—recruiting, NCT03850067).

#### AZD5153

A novel, reversible BRD4 inhibitor endowed with bivalent mechanism of action and substantial antitumor potential as evidenced in preclinical studies. In a phase 1 dose escalation study, oral AZD5153 QD/BID was administered to patients with RR solid tumor, including lymphoma. 28 patients were treated in 7 cohorts. The results of the study demonstrated that treatment related AEs were observed in 50% patients. Moreover, a linear increase in PK was evidenced. Overall, it was concluded that AZD5153 as a single agent is safe and well tolerated (30 mg QD and 15 mg BID) (NCT03205176) [304] AZD5153 is also undergoing phase 1 studies in combination with Acalabrutinib in patients with R/R aggressive NHL (NCT03527147).

#### FT-1101

A promising pan-BET inhibitors possessing equipotent inhibition for BRD2, BRD3, BRD4, and BRDT. FT-1101 exerts substantial anti-proliferative effects against a panel of human leukemia cell lines. FT-1101 has demonstrated higher tumor growth inhibition (TGI) in xenograft and syngeneic models in a relative comparison with JQ1 [305, 306]. Phase 1 clinical trial of FT-1101 (monotherapy and in combination with Azacitidine) in patients with R/R hematologic malignancies got completed in March 2019. [NCT02543879]. The study involved the administration of oral FT-1101 (10–600 mg) dosed once a week, every other week or monthly during dose escalation. The results of the study indicated that 1 patient (out of the 30 evaluable patients) on the 400 mg (every other week) schedule showed complete remission with incomplete hematologic recovery and 19 pts attained SD. Only one patient among the evaluable NHL patients (n = 3) achieved SD. Overall, FT-1101 was found to be safe and exhibited acceptable PK and modest clinical activity in R/R AML/MDS and NHL pts [306] (NCT0254387).

#### PLX51107

An orally active small molecule inhibitor that blocks interactions mediated by the four BET family proteins at low nanomolar potency [41]. A study of PLX51107 (3 + 3 dose escalation study, phase I) was conducted in subjects with R/R solid tumors (lymphomas included) and AML. The study design and results are as follows: study type: 3 + 3 dose escalation study (Phase 1) type of administration: oral, continuous (QD and BID), number of patient enrolled = 36, common AEs (in ≥ 15 pts)—fatigue (33%), vomiting (25%), diarrhea (25%), nausea (19%), bilirubin increase (17%) and INR increase (17%); Results: 8 patients out of 36 pts achieved SD (2 uveal melanoma, 3 sarcomas, 1 CRPC, 1 NSCLC) ranging 4–14 months [307] ((NCT02683395). The study has been terminated owing to business related reasons. The phase 1 combination studies (PLX51107 and Azacitidine) for the treatment of patients with AML or MDS to determine the minimum safe and biologically-effective dose was started on September 2019. (Status—recruiting, NCT04022785).

#### SF1126

SF-1126 is a dual inhibitor of phosphatidylinositol-3-kinase and BRD4. It exerts simultaneous disruption of two key MYC-mediating factors that promote cancer cell growth (NCT03059147). SF1126 was administered IV days 1 and 4, weekly in 28 day-cycles in phase I study in subjects (n = 44) with advanced solid tumours and B-cell malignancies. The results of the study are as follows: toxicity: grade 1 and 2 toxicities with a single DLT at 180 mg/m^2^ (diarrhea); best response: stable disease in 19 of 33 (58%) evaluable patients; maximum administered dose (1110 mg/m^2^). Overall, SF1126 was well tolerated [308]. A phase 1 study of SF1126 with R/R neuroblastoma was terminated due to low patient accrual (NCT02337309). The clinical evaluation of SF1126 in combination with nivolumab in patients with advanced HCC was initiated on March 27, 2017 (status—active not recruiting, NCT03059147). A phase II Study SF1126, in patients with recurrent or progressive SCCHN and mutations in PIK3CA gene and/or PI-3 kinase pathway genes was terminated due to slow enrolment (NCT02644122).

## Medicinal chemist’s perspective: prudent approaches to steer the progress of the epigenetic inhibitors from preclinical to clinical level

The clinical advancement of numerous epigenetic inhibitors as discussed in the preceding sections coupled with the better understanding and growing insights in context of the role of epigenetic mutations in cancer clearly renders ample scope for the medicinal chemist to expand the size of the armoury (epigenetic inhibitors) at the preclinical level. On the precedential basis, it has been well evidenced that only a few preclinical active scaffolds are able to replicate their promising activity profile at the clinical level. Thus it becomes imperative to have a sagacious understanding of drug design at the root level for rationally constructing epigenetic inhibitors employing diverse strategies. Much to the delight of the medicinal chemist, most of the epigenetic targets are druggable and small molecules have been exploited exhaustively to bind to these targets in pursuit of attaining therapeutic benefits in cancer. In this section, we discuss the potential structural engineering approaches citing several selected literature precedents that appears to be promising to push the preclinical pipeline of the epigenetic drugs to the clinical level by leaps and bounds. The furnishment of new inhibitors is most likely to be achieved through the below mentioned pragmatic approaches:

### Proteolysis-targeting chimera “PROTAC”

**Fig. 8:**
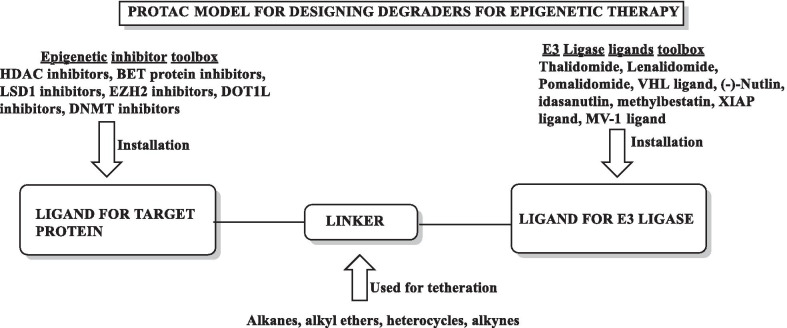
PROTAC design

Growing inclination towards the development of enzyme/protein degraders appears to be most pragmatic way to carry forward the growth of epigenetic therapy. Literature precedents reveals that the degradation of the target protein can yield enhanced therapeutic benefits at low concentrations and thus futuristic attempts should be majorly inclined towards the development of epigenetic inhibitor based PROTACs. As such, PROTACs are hetero bifunctional small molecules composed of three chemical elements namely ligand for the target protein, ligand for E3 ubiquitin ligase and a linker for the tetheration of the two ligands (Fig. 8) [309]. Engineered to induce degradation of disease causing proteins by ubiquitin proteasome pathway, PROTAC degrade disease-causing proteins through the cell’s ubiquitin/proteasome system and functions by recruiting an E3 ligase to tag the target protein for ubiquitination. Gratifyingly, PROTACs can induce degradation of the target proteins at low exposures and are catalytic in their mode of action [309–311].

**Fig. 9:**
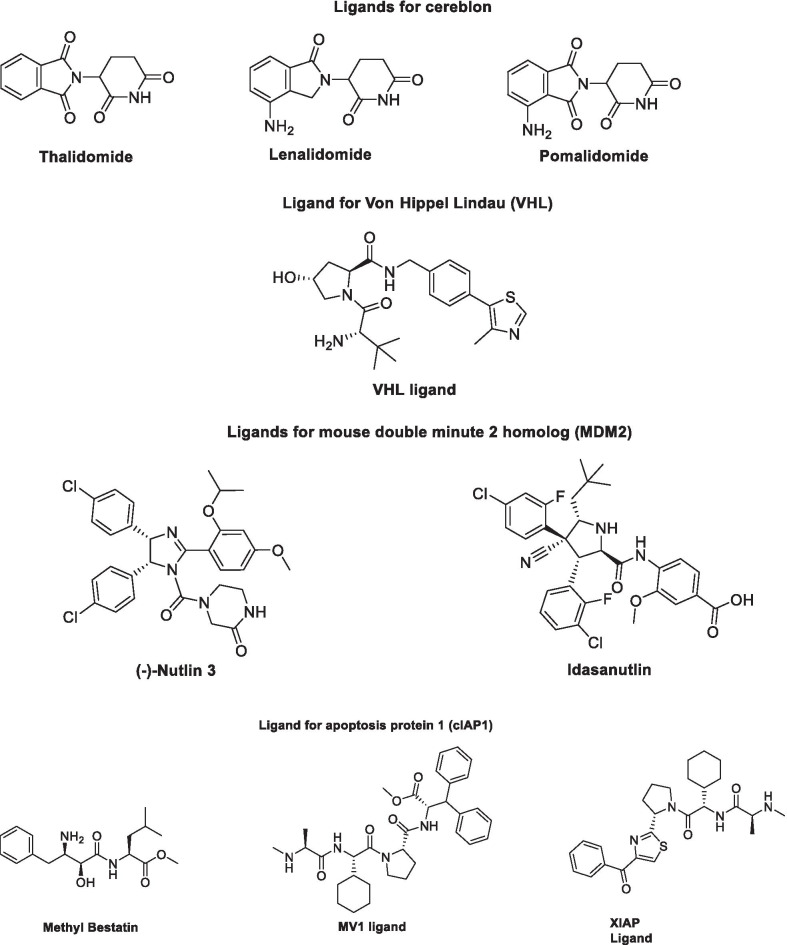
Toolbox of ligands for E3 ligases

Epigenetic drug discovery field, at present, is flooded with numerous agents that have shown promise at preclinical or early stage clinical level. An obvious step forward to extract the benefits of these inhibitors is their installation in the PROTAC model as ligands for the target protein (Fig. 8). Most classes of epigenetic inhibitors consist of simple chemical architectures that offer ample scope to tether them to the ligand for the E3 ligase through a linker. Moreover, epigenetic targets comprise of a wide range of enzymes (HDAC, LSD1, DOT1L, EZH2, BET proteins, DNMT) and this fact can be leveraged for the design of libraries of PROTAC based scaffolds that can demonstrate amplified antiproliferative effects. Practically, quest to accomplish epigenetic inhibitors based PROTACS will require a selection of a target followed by recruitment of a structure (ligand for the target protein) that not just is efficacious in terms of binding to an enzyme/protein or receptor but also bears a suitable site for tetheration with the E3 ligase ligand via a linker. The recruitment of ligand for the E3 ligases is a difficult task as there are limited options (Fig. 9) available in context of the chemical architectures. Usually, the expression pattern of E3 ligases in a particular tissue (Table 5) is observed to select the ligand for E3 ligase. For instance, high expression of E3 ligase CRBN is reported in lung cancer, thus ligands for CRBN (lenidomide, thalidomide, pomalidomide) should be installed in the PROTAC model to develop an anti-lung cancer therapeutic. Likewise, VHL ligand requires to be accommodated in the PROTAC template to target the MDM2 E3 ligase that is overexpressed in liver cancer [312]. Forth this, attention needs to be directed towards the selection of linker which connects two functional heads: a ligand for E3 ligase recognition and a ligand for target protein recognition. Linker is also a crucial element of the structural template of PROTACS and plays a key role in efficient ubiquitination of the target protein and its ultimate degradation. The available literature reveals that linker represent the most variable vector of this model and researcher have exploited diverse chemical functionalities as linkers (synthetically tractable alkyl chains, polyethylene glycol, heteroaryl linkers generated via click chemistry) to afford the formation of the PROTAC ternary complexes. The chemist usually varies the length of the linker to fine tune the distance between two participating partner proteins in pursuit of attaining efficient degradation, thereby extracting enhanced therapeutic benefits. To add on, maximal binding affinity can also be attained by optimizing the linker position to afford various derivatizations. It is noteworthy to mention that the linker part is optimized not only for the achieving potent bioactivity but also for rendering appropriate physicochemical properties to the resulting scaffolds. Literature precedents reveals that limited attention has been paid so far towards the linker design, however, the evidenced increasing interest of the researchers towards the design of PROTACS is expected to expand the size of the linker library by exploration of sophisticated functional linkers [309–311]. Installation of diverse linkers will enable the chemist to ascertain the impact of subtle and unsubtle structural variations in the chemical nature of linker on the anticancer profile of the PROTACS, ultimately generating the structure activity relationship.

**Table 5:** Tissue expression of frequently recruited E3 ligase in PROTAC design [312]

E3 ligases	Tissue expression of E3 ligases
CRBN	Highly expressed in adrenal gland, appendix, bronchus, cerebellum, cerebral cortex, colon duodenum, heart muscle, liver, lung, lymph node, oral mucosa, pancreas, parathyroid gland, placenta, salivary gland, soft tissue 1, thymus thyroid gland, tonsil, vagina
MDM2	Highly expressed in adrenal gland, appendix, bone marrow, breast, bronchus, caudate, cerebellum, cerebral cortex, cervix uterine, colon, duodenum, endometrium, epididymis, oesophagus, eye, gall bladder, hippocampus, heart muscle, kidney, liver, lung, lymph node, nasopharynx, oral mucosa, ovary, pancreas, parathyroid gland, placenta, prostate, rectum, salivary gland, seminal vesicle, skeletal muscle, skin, small intestine, smooth muscle, soft tissue 1, soft tissue 2, spleen, stomach, testis, thymus, thymus thyroid gland, tonsil, urinary bladder, vagina
XIAP	Highly expressed in adrenal gland, heart muscle, hippocampus, seminal vesicle, tonsil
VHL	High expressed in gall bladder, kidney Moderately expressed in salivary gland, liver, pancreas, epididymis,

Once each component of the PROTAC model is finalized, a site of tetheration on the ligand for the target protein and ligand for E3 ligase is identified either through the previously established structure activity relationship or through the docking study of the ligand with in the active site of target protein. Forth this, a synthetic route is proposed that requires an in-depth knowledge of classical organic chemistry concepts. For the tetheration of components, click chemistry (azide-alkyne cycloaddition), organometallic chemistry (Heck coupling, sonogoshira coupling, stille coupling, suzuki coupling, negishi coupling, Buchwald–Hartwig coupling) and amide coupling methodologies (EDC/HOBT, DCC, TBTU, HBTU, PyBOP mediated) have been most exhaustively utilized. In comparison to other therapeutic modalities, PROTAC offers significant advantages such as elimination of target proteins, good oral availability, ease of attaining high potency/selectivity at nonomolar or even picomolar concentrations and high tissue penetration [309–311, 313].

**Fig. 10:**
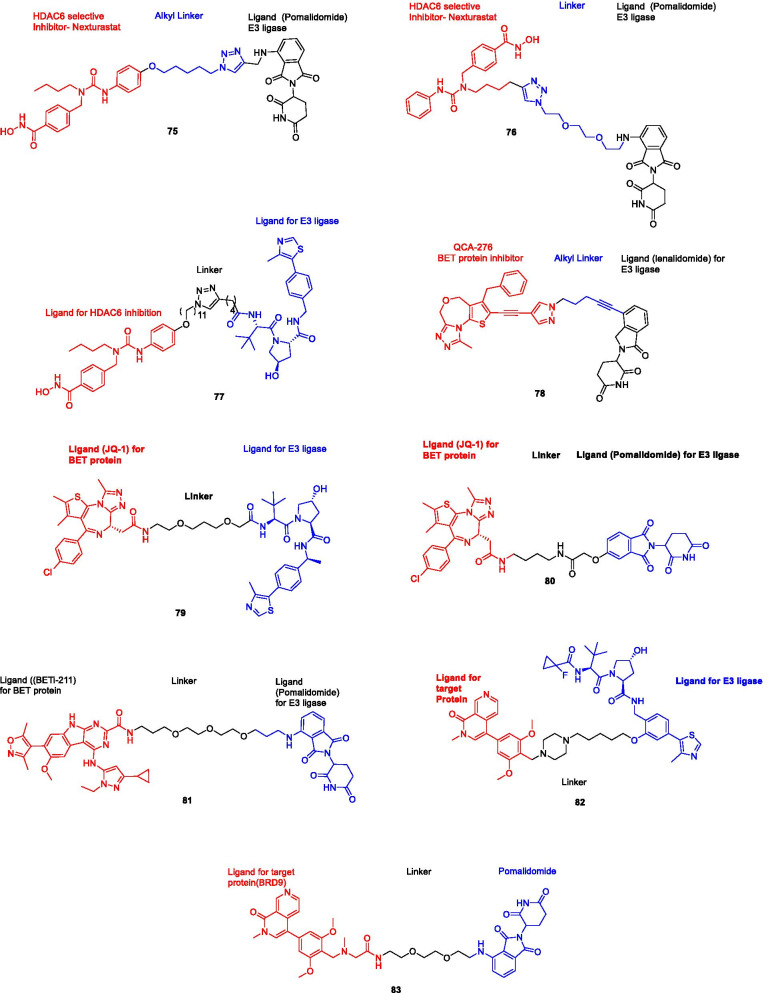
Epigenetic inhibitor based PROTACs

More recently, epigenetic inhibitors have garnered significant attention of researchers in pursuit of degrading the target protein and this has led to the initiation of several explorations for the development of epigenetic inhibitor installed PROTACs (Fig. 10). Some of the promising studies have been covered in this section.

#### Small-molecule HDAC degraders (PROTAC)

Wu et al. reported multifunctional HDAC6 degraders furnished via tetheration of Nexturastat A, a selective HDAC6 inhibitor, with CRBN ligand. Variation of the linker length as well as the linking position culminated in the series of compounds that were evaluated for their HDAC6 degradation effects along with efficacy in multiple myeloma. The results of the study led to the identification of a PROTAC **75** that demonstrated striking selectivity as well as potency and selectivity for the HDAC6 degradation, clearly evidenced with the upregulated levels of HDAC6. The PROTAC also demonstrated promising antiproliferative effects in multiple myeloma cells [314]. Installation of nexturasat in the PROTAC model was attempted in another study performed by Rao et al. Resultantly, a potent PROTAC **76** was identified that exerted significant reduction in the levels of HDAC6 protein level (HeLa cells) at a concentration of 100 nM [315]. Yang et al. recently reported HDAC6 degraders via recruitment of VHL (ligand for E3 ligase) instead of CRBN and identified an extremely potent PROTAC **77** as HDAC6 degrader. Overall the results of the study were highly optimistic and indicated that PROTAC can be utilized as a specific chemical probe for HDAC6 degradation to further investigate HDAC6-related biological pathways (Fig. 10) [316].

#### Small-molecule BET degraders (PROTAC)

Previously conducted structure-guided design of [1,4] oxazepines by Qin et al. led to the identification of QCA276 that was used as a starting point for the design of small-molecule BET degraders (PROTAC). The degraders were synthesized and subsequently evaluated for BET degradation. Owing to the exhaustive pharmacological explorations, one of the compound, QCA570 (**78**) emerged as an extremely potent BET degrader **78** that demonstrated BET proteins degradation along with cell growth inhibitory activity towards human acute leukemia cell lines at low picomolar concentrations. In leukemia xenograft models, complete and durable tumor regression was attained by the use of QCA570 (**78**) at well-tolerated dose-schedules [317]. Crew et al. reported ARV-771 (**79**) as a pan-BET degrader that caused degradation of c-MYC and induced apoptosis of cells through PARP cleavage. The degrader was evaluated in vivo employing the VCaP tumor model and it was observed that the degrader could exert significant tumor growth inhibition. On the whole, ARV-771 (**79**) demonstrated high potential for treating CRPC compared to enzalutamide [318]. Bardners et al. furnished a BET degrader via conjugation of JQ1 and pomalidomide. The resulting degrader **80** displayed substantial degradation potential towards BRD4 and could also inhibit the growth of tumor as evidenced in an in vivo murine hind-limb xenograft model with human MV4-11 leukemia cells [319]. Wang et al. designed a BET degrader **81** using a previously reported BET inhibitor (BETi-211) for the treatment of TNBC. The degrader targeted BRD2, BRD3 and BRD4 in a dose-dependent manner and inhibited a TNBC cell growth at nanomolar concentration. A time dependent downregulation of MCL1 protein was also exerted by the degrader. Moreover, in the patient-derived xenograft model of TNBC, the degrader demonstrated high efficacy [320]. A BRD9 degrader was generated via conjugation of the VHL ligand and a BRD9 inhibitor by cullin et al. The degrader **82** was found to be high efficacious as it could induce the degradation of BRD9 and BRD7 at nonomolar concentrations. The CRBN based PROTAC also displayed remarkable cytotoxic effects against acute myeloid eosinophilic leukemia and malignant rhabdoid tumor [321]. A BRD9 degrader **83** was designed and synthesized by Bradner et al. that could induce the degradation of the target protein at nanomolar concentration and also exhibited more pronounced antiproliferative effects in the human AML MOLM-13 cell line than the inhibitor (BRD 9 inhibitor) used for the construction of the PROTAC assemblage (Fig. 10) [322].

#### Challenges associated with PROTAC approach

In light of the aforementioned, PROTAC appears to be a fascinating stratagem that holds tremendous potential to save the sinking ships of epigenetic inhibitors particularly the ones that are considered to be failures as single agents and are being only considered as suitable candidates for combination therapy. Albeit, endowed with significant merits, PROTAC approach also poses some challenges that need to be addressed to extract a cancer therapeutic out of it in the longer run. At present, it is reported that an estimated 600 E3 ligases have unique activity profiles and distribution patterns throughout the body, however only a limited number of ligands have been identified for them. Thus an exhaustive screening program is required to be initiated for identifying the ligands for E3 ligases as picking the right ligase to tag the target protein is extremely imperative for the success of this program. Other than this, no obvious explanation has been provided so far in the majority of the reported studies behind the rational for selection of the linker and it appears a random selection of linkers is usually made. In this context, a more pragmatic inclusion of the linkers is required to ascertain conclusive benefits of this approach. In addition, to unleash the true potential of PROTACs, comprehensive explorations are required to be conducted at the clinical level to gain deeper mechanistic insights of the PROTACs that have passed the preclinical stage.

### Multitargeting agents

The concept of multitargeting approach is not a new concept and numerous hybrid scaffolds composed of more than one pharmacophore to exert concomitant modulation of two or more targets have been successfully furnished in the last decade. Indeed, epigenetic inhibitors field should be duly credited for the regained attention towards the scaffolds that are promiscuous and were once considered to be “not the first choice” of the medicinal chemist. Usually a biochemical relation between the two targets or the optimistic results demonstrated by a cocktail of drugs lays the foundation for the construction of such scaffolds. Interestingly, this strategy confers a broad platform to the chemist for the generation of logically constructed scaffolds with enhanced antitumor effects. It is noteworthy to mention that this approach has particularly capitalized on the flexibility of the three component HDAC inhibitory pharmacophore and thus the pipeline of multitargeting epigenetic inhibitors is flooded with the agents that inhibit HDAC isoforms along with the other biochemically correlated target. Moreover, the approach has also been extended towards non-epigenetic target and numerous hybrid scaffolds decorated with one fragment from epigenetic inhibitors and the other fragment form epigenetic or non-epigenetic inhibitor are reported. Decades of extensive research indicates that the chemist has displayed utmost proficiency for rationally designing dual epigenetic inhibitory agents and few representative studies that excellently exemplifies this concept are presented below:

#### Dual HDAC-HSP90 inhibitor

**Fig. 11:**
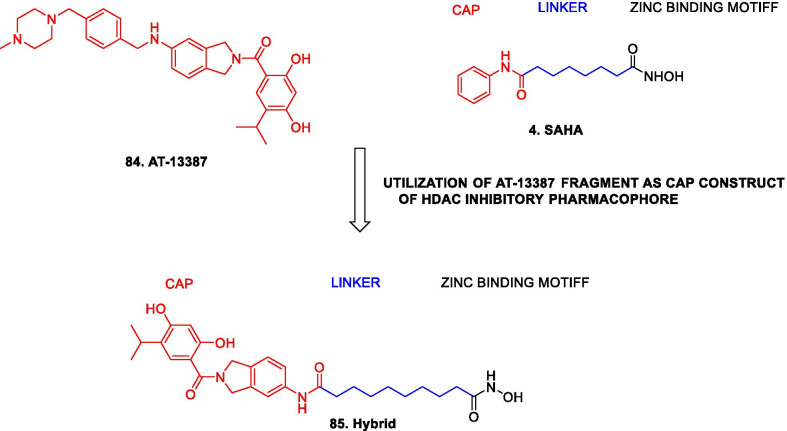
Design of dual HDAC-HSP90 inhibitor

Synergistic anticancer efficacy attained with HDAC and HSP90 inhibition coupled with the evidenced ability of HDAC inhibitors to induce acetylation and inhibit the ATP binding and chaperone function of HSP90 protein provides a strong rationale for the fabrication of dual HDAC-HSP90 inhibitor [323–329]. To add on, revelations ascertaining that the beneficial effects that can be attained via targeting of the HDAC6/HSP90 Axis in NSCLC [330] further strengthens the logic behind the design of hybrid scaffolds (**85**) composed of pharmacophoric features of HDAC as well as HSP90 inhibitors. With this background, a hybrid scaffold was furnished bearing the key structural units of a second generation HSP90 inhibitor, AT-13387 (**84**) and FDA approved hydroxamic acid, SAHA (Fig. 11). The results of the in-vitro and in-vivo studies demonstrated that the resulting adduct (**85**) could remarkably inhibit the HDAC6 isoform (IC_50_ = 4.3 nM) and HSP90 protein (IC_50_ = 46.8 nM) and could also exert substantial antiproliferative effects against the NSCLC (A549 and H1975). Expressions of signatory biomarkers associated with HDAC6 and HSP90 inhibition were also modulated by the hybrid compound (**85**). Other than the striking in-vitro antiproliferative profile, the hybrid scaffold was also endowed with tumour growth inhibitory potential as evidenced in human EGFR-resistance NSCLC H1975 xenograft model in vivo [331]*.*

#### Dual HDAC-DNMT inhibitors

**Fig. 12:**
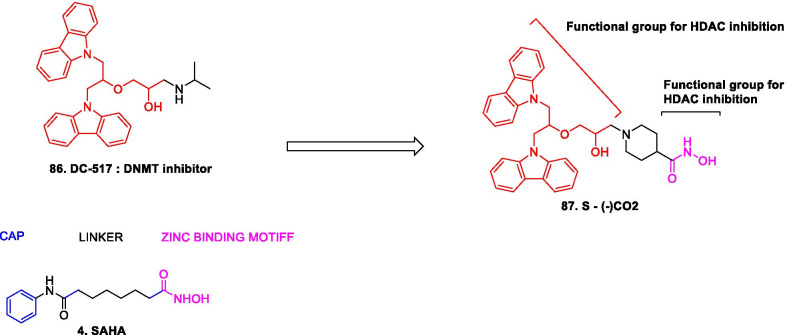
Design of dual HDAC-DNMT inhibitor

Motivated by the optimistic results attained with a cocktail of DNMT and HDAC inhibitor in context of the anticancer efficacy including the suppression of the tumorigenicity of cancer stem-like cells and enhancing cancer immune therapy [332–336], Yuan et al. designed and synthesized a dual DNMT and HDAC inhibitor C02S (**87**) (Fig. 12) that demonstrated significant enzymatic inhibitory activities against DNMT1, DNMT3A, DNMT3B and HDAC1. It was quite evident from the results that the hybrid compound could also inhibit DNMT and HDAC at cellular levels via inversion of mutated methylation and acetylation and increased expression of tumor suppressor proteins. Detailed investigation of C02S revealed that it could induce reexpression of p16, p21 and TIMP3 and cause DNA damages, modulate multiple cancer hallmarks simultaneously and exert tumor growth suppression in mouse breast cancer models [337].

#### Dual HDAC-LSD1 inhibitors

**Fig. 13:**
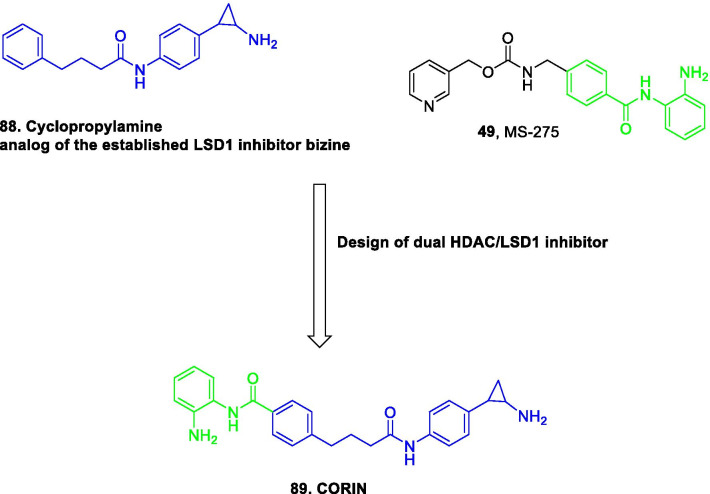
Design of dual HDAC-DNMT inhibitor

Literature precedents underscores the association of gene expression silencing with HDAC1/2 and LSD1 enzymatic activities within the CoREST complex (HDAC complex that includes HDAC1, HDAC2, the scaffolding protein CoREST, and LSD1) that contributes to cancer and other diseases [338, 339]. These notions spurred a group of researchers to design dual LSD1/HDAC inhibitors (Fig. 13) anticipating that such constructs might demonstrate enhanced activity coupled with an improved therapeutic window. The results of the study led to the identification of a dual inhibitor, corin, endowed with magnificent anti-proliferative activity against several melanoma lines and cutaneous squamous cell carcinoma lines. It is noteworthy to mention that the dual LSD1/HDAC inhibitors displayed more pronounced efficacy than its parent monofunctional inhibitors. Detailed investigation of corin (**89**) revealed that its striking pharmacological profile relied on an intact CoREST complex. In the melanoma mouse xenograft model, treatment with corin (**89**) resulted in slowing of the tumor growth [340].

#### Dual HDAC-BET protein inhibitors

**Fig. 14:**
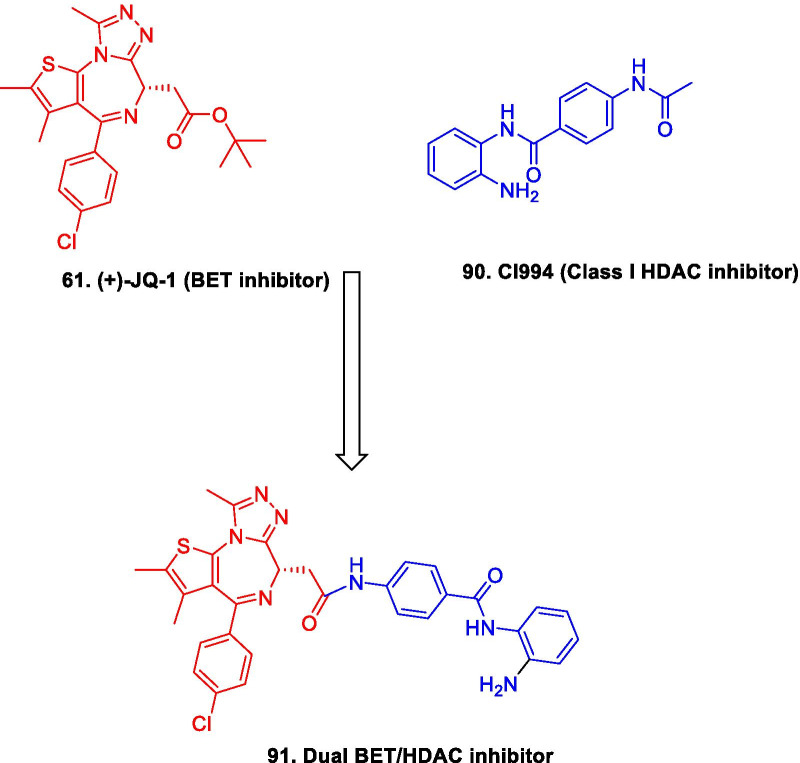
Design of dual HDAC-BET inhibitor

In light of role of BET and HDAC proteins as central regulators of chromatin structure and transcription coupled with the evidenced efficacy attained with the combined BET and HDAC inhibition in pancreatic ductal adenocarcinoma at the preclinical level [341], a hybrid scaffold composed of the structural features of a BET inhibitor ( +)-JQ1 and class I HDAC inhibitor CI994 was generated (Fig. 14). Remarkable tumor cell proliferation was achieved with the hybrid compound in comparison to ( +)-JQ1, CI994 alone or combined treatment of both inhibitors. The hybrid scaffold (**91**) demonstrated more pronounced inhibition of HDAC1 isoform and retained a similar inhibitory potency against BRD4 bromodomains as that of ( +)-JQ1. [342]

#### Dual HDAC-EZH2 inhibitors

**Fig. 15:**
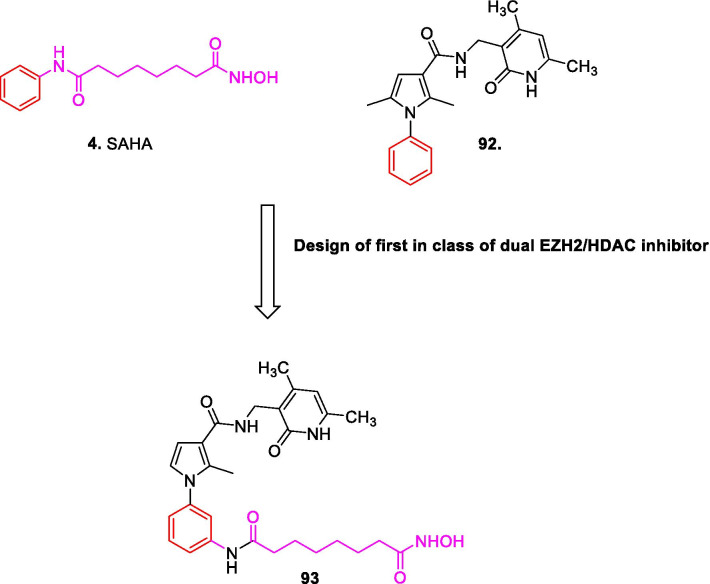
Design of dual HDAC-EZH2 inhibitor

Previous reports reveal that EZH2 works in tandem with HDACs in the same protein complex and mediates gene transcription repression by increasing histone H3 Lys [343–348]. Owing to this functional link, design of dual EZH2/HDAC inhibitor has been conceived as a rational approach to control a number of epigenetic-dependent carcinogenic pathways. In an attempt to capitalize on this useful information, a group of researchers led by Romanelli et al. designed a first-in-class dual EZH2/HDAC inhibitor (**93**) (Fig. 15) that displayed a balanced inhibitory potential towards both the targets and also inhibited the proliferation of U937, THP1 (hematological malignancies) RH4 (rhabdomyosarcoma), SH-N-SK (neuroblastoma) and U87 (glioblastoma) cancer cell lines. Moreover, in U937 and RH4 cells, the dual inhibitor caused cell cycle arrest in the subG1 phase, induced apoptosis and increased the expression of cell differentiation markers [349].

#### Dual HDAC-PI3K inhibitor

**Fig. 16:**
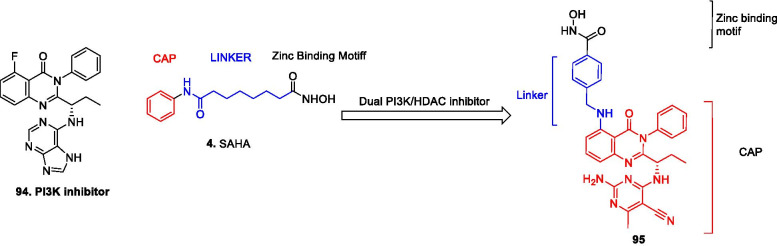
Design of dual HDAC-PI3K inhibitor

In pursuit of attaining synergistic effects from simultaneous inhibition of PI3K and HDAC, Thakur et al. designed quinazolin-4-one based hydroxamic acids using a rational approach for the tetheration of the pharmacophores of both the inhibitors (Fig. 16). Resultantly, some of the hybrid adducts were found to be potent as well as selective against selective against PI3Kγ, δ and HDAC6 enzymes. The dual inhibitors also exhibited cell growth inhibitory effects exhibited against multiple cancer cell lines. One of the most promising dual inhibitor induced necrosis in several mutant and FLT3-resistant AML cell lines and primary blasts from AML patients and was not found to be toxic. The hybrid compound (**95**) was also endowed with a good pharmacokinetic profile when evaluated in mice via imp administration. [350]

#### Dual HDAC-tubulin

**Fig. 17:**
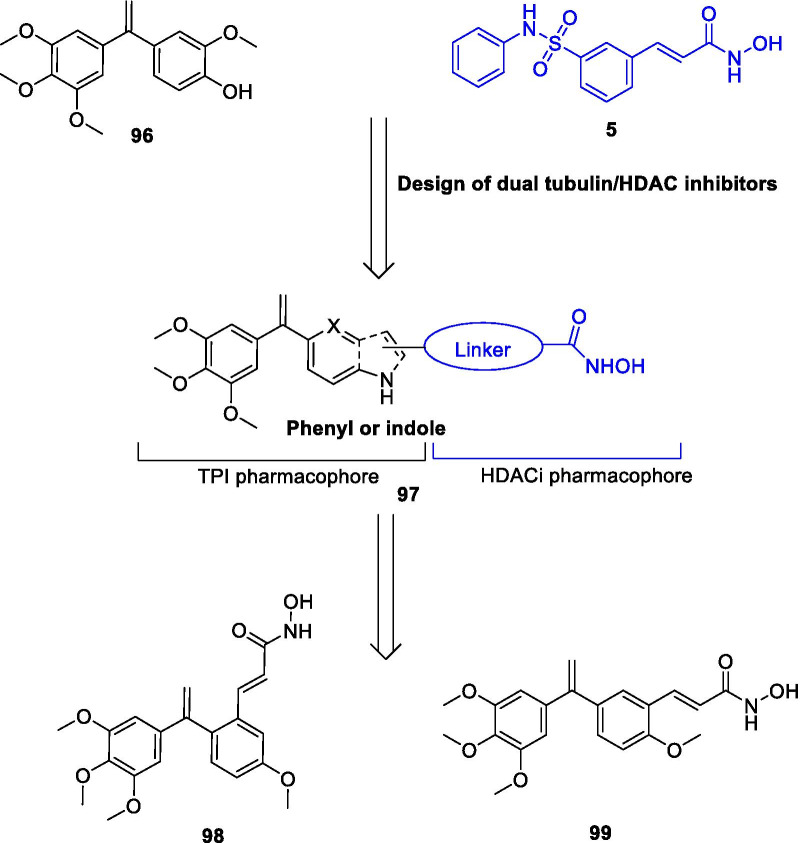
Design of dual HDAC-tubulin inhibitor

An investigation conducted for evaluating the efficacy of vincristine (microtubule destabilizing agent) and vorinostat (HDAC inhibitor) reported that the cocktail of the aforementioned drugs demonstrates synergistic antitumor effects in vitro and in vivo. These promising results were basically attributed to the alteration of the microtubules dynamics via vorinostat exerted HDAC inhibition [351]. Motivated by these findings, dual inhibitors of tubulin polymerization and HDAC were designed by Lamaa et al.employing the key pharmacophoric features of 1,1-diarylethylenes (isoCA-4) and belinostat (Fig. 17). Subsequent evaluation of the dual inhibitors revealed that two of the inhibitors (**98** and **99**) were endowed with striking antiproliferative activity mediated through substantial inhibition of tubulin polymerization as well as HDAC8. One of the compounds, **99**, could induce cell cycle arrest of cancer cells at the G2/M phase via disruption of microtubule organization. Furthermore, docking study also rationalized the binding of these hybrid molecules with both tubulin and HDAC active sites. The compound **99** also exhibited cell growth inhibitory effects against tumoral cell lines such as K562, PC3, U87, and BXPC3 and was also active against the CA-4 refractory human colon adenocarcinoma cell line HT-29 and possessed acceptable physicochemical properties [352].

The aforementioned studies perspicuously highlight that this approach holds substantial promise and optimism and its implementation is expected to be continued to shoulder the progress of new scaffolds as inhibitors of the epigenetic targets. It is evident that single molecule multiple targets”, “multiple ligands” or “hybrid” anticancer agents can enhance efficacy and lower drug resistance as multiple cross talks between the signaling networks are involved in cancer. The dual inhibitors also score over the strategy of using the cocktail of drugs (combination therapy) as they eliminate the need of extensive investigations such as dose limiting toxicity, drug-drug interactions, pharmacokinetics, bioavailability, establishment of the dosage regimen that, as such, are required to be conducted for combination therapy [353]. Endowed with the benefits of lowering the risk of possible drug interactions, simplified drug metabolism, improved drug transport and reduced drug R&D costs, it is anticipated that the multitargeting agents might outshine the candidature of combination therapy for the treatment of cancer. The main challenge in front of the medicinal chemist is to furnish assemblage that can exert balanced modulation of both the targets to produce synergistic antiproliferative effects and this can only be accomplished via careful selection of targets, pharmacophores as well as the site of tetheration.

### Isoform selective inhibitors: Employing a structural template to furnish selective isoform inhibitors of the enzymes

Selecting an epigenetic target for exploration of chemical entities only leads to partial accomplishment of the task as there is still a galactic challenge in front of the medicinal chemist to design isoform selective inhibitors of the enzyme. In general, selective isoform inhibitors are conceived to be less toxic and more efficacious than the non-selective ones. In this context, the ongoing wave in the field of epigenetic drug discovery filed is heavily inclined towards the construction of selective isoform inhibitors to attain anti-tumor effects. This scenario can be best explained by the consideration of the recent trends in the HDAC inhibitors field. As such, HDAC inhibitors garnered limelight with the discovery of pan-HDAC inhibitors namely vorinostat, romidepsin, belinostat, and panobinostat that were later approved by FDA for treatment of diverse malignancies [67–70]. Despite the clinical success evidenced with the pan HDAC inhibitors, their use has been associated with some side effects, such as fatigue, diarrhea, nausea, QTc-interval prolongation and thrombocytopenia [354–357]. These disappointing revelations spurred the researchers to draw their attention towards selective inhibitors of HDAC isoforms. Attaining isoform selectivity via structural engineering approaches appears to be extremely feasible for the HDAC inhibitors in light of their flexible and modular structural template composed of three parts: CAP-Linker-Zinc binding motif. The efforts invested in the past in this context have been delved into the below mentioned categories and some selected examples are discussed.

**Fig. 18:**
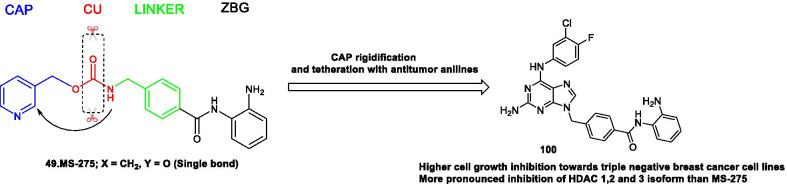
CAP rigidification approach

*CAP modification* The modular nature of the HDAC inhibitory pharmacophore allows the chemist to fine tune each component to attain an inhibitor with a selective isoform inhibitory profile. For instance, a cap construct tolerates a wide variety of modifications such a placement of aryl/heteroaryl ring (bicyclic/tricyclic as well as fused rings), cycloalkanes, bridgehead adducts, spirocyclic rings, steroidal/terpenoidal framework and so on. Other than the placement of diverse scaffolds as CAP component, literature survey indicates that the chemist has also employed several strategies to modify the cap construct such as structural simplification approach, site translocation and CAP rigidification or confernment of some flexibility to the surface recognition part (CAP). Recently, one of the study conducted in our laboratory attempted lead modification of MS-275 employing a CAP rigidification approach that culminated in the identification of a potent compound that was not only a more potent inhibitor of the class I HDACs but was also endowed with substantial antiproliferative effects against TNBC (Fig. 18) [358].

**Fig. 19:**
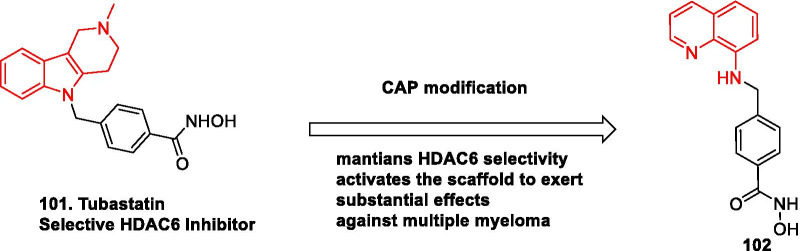
CAP modification

It is noteworthy to mention that the CAP component remains to be the most comprehensively explored part of the HDAC inhibitory pharmacophore not just in pursuit of attaining isoform selectivity but also for induction or amplification of antitumor effects. The HDAC inhibitory pipeline, at present, is also endowed with some candidates that are potent as well as selective isoform inhibitors of the enzyme but are unable to produce anti proliferative effects. Tubastatin, a highly selective HDAC6 inhibitor, exemplifies one of such case where the selective HDAC6 inhibition is just able to induce neurodegenerative effects irrespective of that fact that HDAC6 isoform is overexpressed in various malignancies. In this context, a study was conducted by our research group to modify the cap construct of tubastatin (structure simplification approach) and the resulting compounds displayed excellent activity profile against multiple myeloma coupled with a striking HDAC6 inhibitory profile (Fig. 19) [359]. Thus, the role of CAP construct also appears to the crucial for activating an enzyme inhibitor to demonstrate cellular potency.

**Fig. 20:**
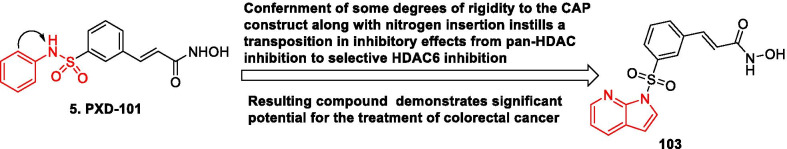
CAP modification

The surface recognition part has also demonstrated scope for structural alteration to afford a transposition in the pattern of inhibitory effects from pan-HDAC inhibition to selective isoform inhibitors of the enzyme. For example, a study focusing on the structural alteration of PXD-101 reported that conferring few degrees of rigidity to the surface recognition part (CAP) via placement of bicyclic ring (Azaindoles) in the chemical architecture of PXD-101 instilled a transposition in inhibitory effects from pan-HDAC inhibition to selective HDAC6 inhibition. (Fig. 20) [360]

**Fig. 21:**
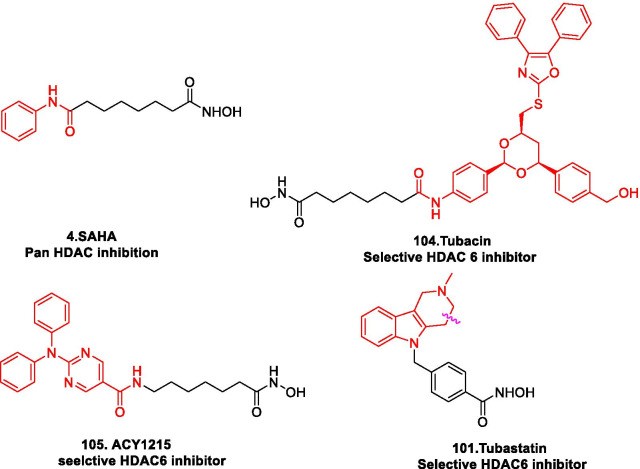
Clinically and preclinically active HDAC inhibitors

*CAP and Linker modification* On similar lines, the linker part has also demonstrated significant accommodative ability and several functionalities have been employed for the design of HDAC inhibitor. Digging the structural architecture of FDA approved inhibitors viz. Vorinostat, belinostat and panabinostat reveals that each of the structure comprises of different type of a linker (long chain alkyl in SAHA, benzyl acrylamide in LBH-589 and benzenesulfonyl acrylamide in PXD-101) yet exerts pan-HDAC inhibition. However, utilization of the same linker of SAHA with an altered CAP construct (chemically bulky) leads to two selective HDAC6 inhibitors namely ACY-1215 [361] and Tubacin [362]. The identification of ACY-1215 and tubacin presents only one example but the fact that diverse combinations of CAP and linkers can be exploited to extract selective isoform inhibition is supported by a plethora of studies. A relative comparison of the template of SAHA with tubacin and ACY-1215 makes it evident that expanding the size of the CAP construct can induce a transposed pattern of HDAC enzyme inhibition from pan HDAC inhibition to selective HDAC6 inhibition. Likewise, taking cognizance of the structural features of another highly potent and selective HDAC6 inhibitor, Tubastatin [363], it can be conceived that a relatively rigid and fused CAP construct requires a change in the chemical nature of linker to exert selective HDAC 6 inhibition (a relative comparison of tubastatin with tubacin and ACY-1215) (Fig. 21).

**Fig. 22:**
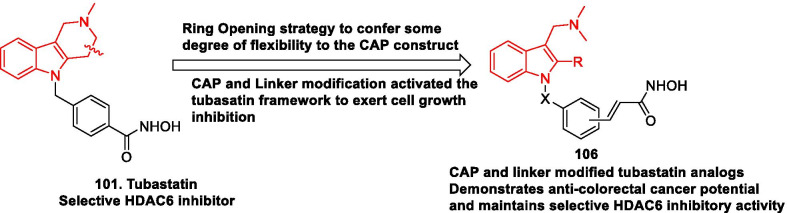
Ring opening strategy

Several structure alteration programs on a lead compound (already reported selective HDAC6 inhibitors) have also been conducted in the past where modifications of cap and linker part were concomitantly attempted. A recent lead modification study on tubastatin excellently exemplifies this case as the rigid CAP construct of tubastatin was modified into ring opened indole ring bearing a flexible dimethyl amino substituent via a structural simplification approach and acrylamide unit as present in FDA approved agents PXD-101 and LBH-529 was installed in the linker region. The results of the study were overwhelmingly positive as the structurally modified tubastatin analogs exhibited significant cellular growth inhibitory effects and maintained their tendency to exert preferential HDAC6 inhibition (Fig. 22).

**Fig. 23:**
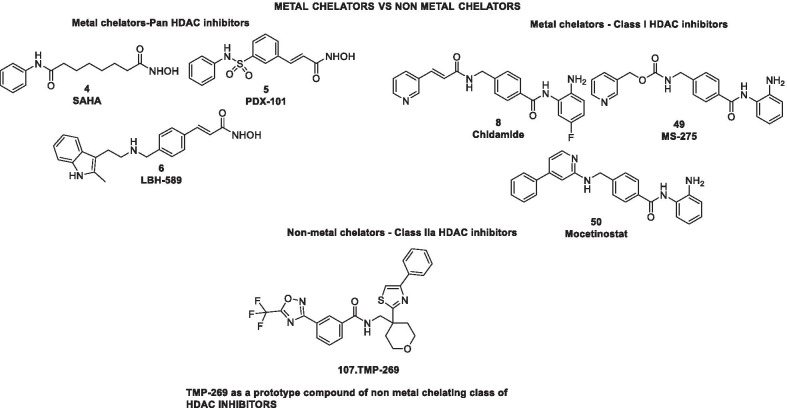
Classification on the basis of zinc binding motif

C. *Modification at the zinc binding motifs* Like the CAP construct and the linker part, selection of the zinc binding motif is also an area that needs tantamount attention to attain selective inhibition of HDAC isoforms, thereby extracting the anticancer effects in malignancies having overexpressed pattern of those isoforms. Practically, the competition is majorly between two classes of zinc binding motif viz. hydroxamic acid and aminoanilides. As such, the former class clearly outshines the latter in terms of clinical success with 3 hydroxamic acid type HDAC inhibitors receiving FDA approval for use in cancer [68–70]. However, chidamide represents the only aminoanilide that has been approved by CFDA to treat patients with recurrent or refractory PTCL [72]. Regardless of the clinical success, the present trend in this field is equally aligned towards the development of both the classes of inhibitors. An obvious explanation to this is the evidenced susceptibility of hydroxamic acids to glucuronide conjugation leading to inactivation and off-targeting whereas some explorations reported that aminoanilides are less prone to glucuronide metabolism and are endowed with high efficacy possibly due to their slow, tight binding and slow disassembling with HDACs [190, 191, 363–368]. Another notable variation between the two classes can be observed in their enzymatic inhibitory profile as pan HDAC inhibition and selective HDAC 6 inhibition is mostly exerted via use of hydroxamic acid based HDAC inhibitors while class I HDAC selectivity is usually attained via the fabrication of aminoanilides. Other than these two types of zinc binding motifs, a trifluoromethyloxadiazolyl moiety (TFMO) as a non-metal chelating group has further led to a categorical division of HDAC inhibitors as the metal chelators and the non-metal chelators. TFMO interacts by one of the fluorine atoms and its oxygen with the active Zn2 + atom in the catalytic center and represents another class of zinc binding motif [369, 370] (Fig. 23). In light of the debated candidature of hydroxamic acids and to some extent, amino anilides also, due to the susceptibility to glucuronidation based inactivation, explorations have been accelerated for non-metal chelating class of HDAC inhibitors in recent times. As a result, TMP-269 has emerged as a prototype inhibitor that bears a trifluoromethyloxadiazolyl moiety (TFMO) as a nonchelating MBG and acts as a class IIa HDAC selective inhibitor [369]. The attributes of TFMO bearing adducts act as boon for the medicinal chemist working in this area as utilization of this zinc binding motif can accomplish HDAC inhibitors that are non-susceptible to glucoronidation and can generate selective/preferential class IIa inhibitory adducts (class IIa HDAC bias). To add on, leveraging the TFMO group for furnishing the HDAC inhibitory assemblage presents a rational approach that might challenge the strategy of targeting a metalloenzyme only with a metal chelators. It is anticipated that conclusive therapeutic benefits can be achieved by shifting the inclination towards weak binders of the metal that are duly supported by other structural features to garner favourable interactions with the amino acid residues of the active site. This aforementioned information clearly provides a platform to pragmatically design new chemical entities that can target the malignancies having an overexpressed pattern of class IIa HDACs.

Overall, the approach to construct HDAC inhibitors will require an in-depth literature survey to extract the information regarding the over-expression of various HDAC isoforms in diverse malignancies and accordingly adducts can be designed to bear carefully recruited components of the HDAC inhibitory model. Consideration of these notions can certainly enable us to create a depository of isoform selective HDAC inhibitors that upon further evaluation such as evaluation of ADME properties, high dose pharmacology, toxicity studies, evaluation in animal models that mimics the human condition can advance to clinical stage investigation.

### Fragment stitching approach: Fragment stitching on an existing drug to produce more potent derivatives

**Fig. 24:**
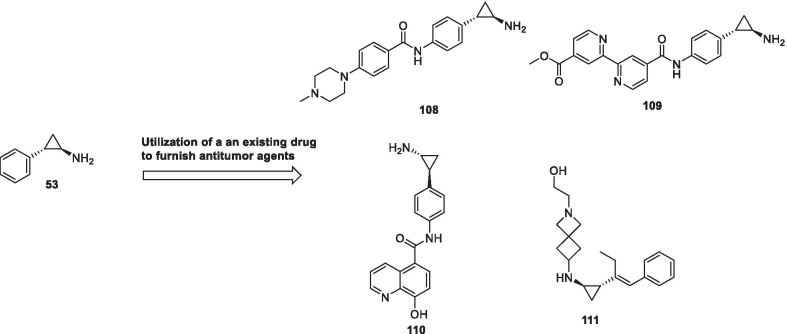
Fragment stitching approach

Unlike the approach of using a structural template to create a compendium of compounds (via diverse permutations and combinations of key components) to target various isoforms of the enzyme, this strategy simply employs a FDA approved drug to stich diverse fragments that either leads to the induction or potentiation of anticancer effects. Usually a transposition in the isoform inhibitory effects is not desired rather inhibition of the same isoform is capitalized to derive antitumour effects in diverse malignancies. This approach can be best explained by the numerous investigations conducted on a LSD1 inhibitor, tranylcypromine, which has been exhaustively utilized in the past to fill the library of rationally designed analogues with anticancer efficacy (Fig. 24). To exemplify this, some selected structures have been presented in Fig. 24 that were generated via stitching of diverse fragments on the tranylcypromine core. Compound **108** was recently reported by Vianello et al. as potent LSD 1inhibitor that displayed substantial activity in the in vivo model (in the murine promyelocytic leukemia model) on oral administration. The results of the study also demonstrated that compound **108** was well tolerated and led to remarkable prolongment of the survival time of the treated mice (35% and 62% at the doses of 11.25 and 22.50 mg/kg, respectively). [371] Rotili et al. conducted an investigation that led to the identification of LSD1 inhibitors (**109** and **110**). The inhibitors induced an increase in H3K4 and H3K9 methylation levels in cells, caused growth arrest and apoptosis in LNCaP prostate and HCT116 colon cancer cells. [372] A study by gehling et al. led to the identification of a highly potent and selective LSD 1 inhibitor, compound **111,** (< 4 nM biochemical, 2 nM cell, and 1 nM GI_50_). Compound **111** exhibited cell growth inhibitory effects in a panel of AML cell lines along with notable antitumor potential in a Kasumi-1 xenograft model of AML at the dose of 1.5 mg/kg once daily (administered orally) [373].

The structures shown in Fig. 24 only represents some of the selected examples that have been furnished using the aforementioned approach, however, the preclinical pipeline of LSD1 inhibitors is endowed with numerous candidates synthesized via fragment stitching approach. It is noteworthy to mention that this approach has been implemented to all classes of epigenetic inhibitors and the main advantage of employing this approach is that substantial amount of information in terms of protocol for synthesis, structure activity relationship as well as the toxicity profile is available and accessible. This information plays a key role in expediting the library generation of compounds and it is conceived that this startegem is likely to be continually employed in the near future to create antitumor assemblages.

**Fig. 25:**
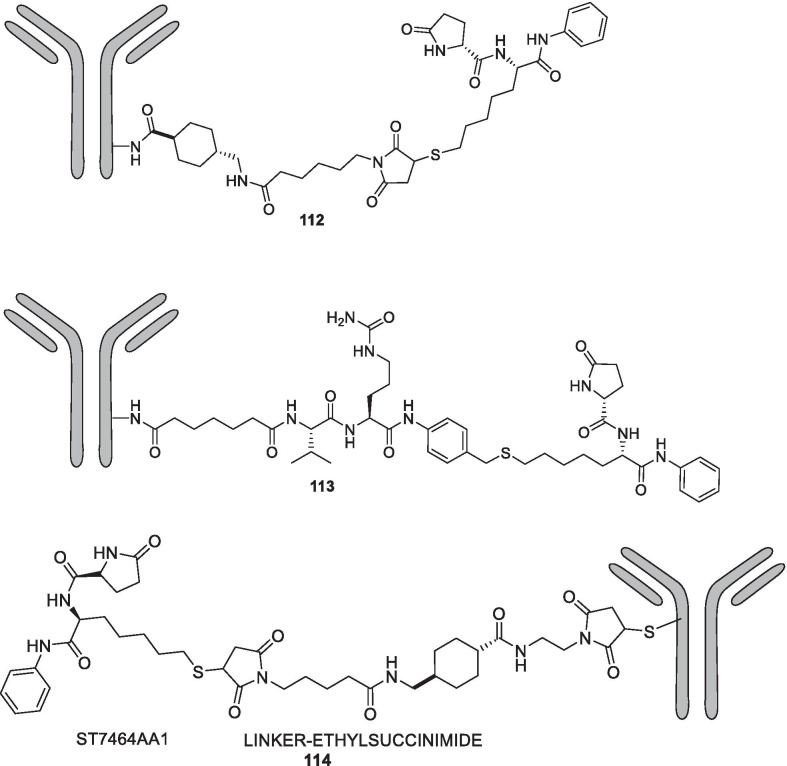
Antibody drug conjugates

*Antibody drug conjugates:* Accomplished via conjugation of a small molecule inhibitor and a humanized antibody though a chemical linker, ADCs selectively bind to the receptors of tumor cells. Internalization of the receptor–ADC complex usually occurs through the endocytosis pathway. Forth the internalization, the cytotoxic drug is released via cleavage of the linker. This strategy of targeted drug delivery presents enough promise to overcome the issue of systemic toxicity and narrow therapeutic window that limits the clinical use of the available chemotherapeutic agents [374–376]. In this context, a study was conducted recently and two antibody-drug conjugates (**112** and **113**) comprising of a HDAC inhibitor ST7612AA1 and cetuximab employing cleavable and non-cleavable linkers were synthesized. The results of the study were extremely promising and the HDAC inhibitor-antibody conjugate demonstrated efficient internalization in tumour cells. In the *in vivo* studies, the conjugates exhibited striking antitumor activity (animal models of human solid tumors) without any toxicity that is generally observed with traditional ADCs delivering highly potent cytotoxic drugs. (Fig. 25) [377]

Another research group recently furnished an ADC endowed with the potential to deliver a HDAC inhibitor to ErbB2 + solid tumors. In the study, partial reduction of trastuzumab with tris [2-carboxyethyl] phosphine was performed followed by conjugation to ST7464AA1, the active form of the prodrug HDAC inhibitor ST7612AA1 via a maleimide-thiol linker to furnish the target ADC ST8176AA1. The detailed biological evaluation of ST8176AA1 revealed a similar receptor binding and internalization of ST8176AA1 to trastuzumab. However, the conjugate demonstrated higher anti-tumor activity compared to trastuzumab (in vitro assay) that correlated with increased acetylation of histones and α-tubulin. Moreover, similar to trastuzumab, ST8176AA1 also increased the expression of ErbB2 and estrogen receptor in TNBC cells. (Fig. 25) [378]

These aforementioned studies provide a strong rational to extend this concept to the targeted delivery of epigenetic inhibitors that, in turn, can demonstrate fruitful results in cancer by exerting epigenetic modulation at a much safer dose in comparison to standard epigenetic inhibitors based therapeutic tools. In this context, it appears logical to dig the preclinical pipeline of the epigenetic inhibitors and select some candidates that could not advance to the clinical stage investigation owing to the toxicity related hindering factors. Once selected, their accommodation in ADC model should be attempted.

### (CRISPR) Cas9

Designing the CRISPR/Cas9-based strategies to target the cancerous epigenetic regulators in a more specific manner is an emerging potential approach that has garnered tremendous interest in the recent past as a tool to correct genetic mutations. CRISPR-based epigenome editors (CRISPR epi-editors) consist of dCas9 and epigenetic effector (fused or non-covalently) [379–382] and are being given serious consideration as a practical approach in cancer gene therapy as they can activate the tumor suppressor genes and also inhibit the tumor driving genes. On the literature precedential basis, it is well known that epigenetic mechanisms sometimes inactivate tumor suppressors in some cancers. To add on, the epigenetic factors, such as LSD1, EZH2 and NSD2 (tumor drivers,) are overexpressed by either epigenetic or genetic mechanisms in diverse malignancies. In light of these revelations, development of CRISPR/Cas9 based transcriptional regulators appears to be a pragmatic way to: (1) suppress the expression of the aforementioned enzymes in some cancers (2) target the driver genes of cancer as well as the genes essential for cancer maintenance or drug resistance (3) target the “undruggable” epigenetically silenced tumor suppressors [379–383]. In a nutshell, dCas9-fused epigenetic regulators holds enough promise for cancer treatment as they can reversibly manipulate the epigenetic patterns and also regulate the oncogenes and tumor suppressors expressions.

## Conclusion and future perspective

Significant advancement has been made in the field of the epigenetic drug discovery in the last decade. All the major classes of the epigenetic tools have received considerable attention. In context of the DNMT inhibitors, the FDA approved DNMT inhibitors 5-azacytidine and decitabine were exhaustively explored in AML and MDS at the clinical level and optimistic results were attained particularly in combination therapy. Other than the FDA approved nucleoside based DNMT inhibitors, guadecitabine, a dinucleotide antimetabolite of a decitabine linked via phosphodiester bond to guanosine and 5-fluoro-2′-deoxycytidine (FdCyd) also demonstrated promise and are likely to be the subjects of number of investigations in diverse cancer types in the near future. In addition, non-nucleoside prototypes namely curcumin, hydralazine, procainamide, disulfiram, SGI-1027 and ((-)-epigallocatechin-3-gal were evidenced to be endowed with interesting antitumor profiles. In light of the current trends in medicinal chemistry, it appears that the balance in context of the future research will be tilted towards the natural product based non-nucleoside inhibitors owing to the beneficial trends evidenced with curcumin coupled with the fact that the chemical architecture of this phytoconstituent comprises of several sites accessible to generate an appropriate sized structure pool. For the EZH2 inhibitory field, several drug candidates are undergoing clinical stage evaluation with GSK126, GSK343, CPI-205, ZLD1039, PF-06821497, UNC1999 representing a few of them. It is noteworthy to mention that the clinical success of tazemetosat has brought the spotlight on EZH2 as a potential target for the design of cancer therapeutics. The studies conducted also indicates that significant benefits can be attained through the simultaneous inhibition of EZH1/EZH2 and thus the dual targeting of the aforementioned is likely to be explored more comprehensively. Moreover, the synergistic effects anticipated to be attained through the combination of EZH2 inhibitors with immunotherapy or chemotherapy are also likely to be evaluated. In context of the preliminary/preclinical exploration, the chemical architectures of tazemetostat offer several sites for chemical alterations that can be exploited to create library of new inhibitors. The area of development of DOT1L inhibitors have relatively received less attention in comparison to the other epigenetic tools and pinometostat, a potent and selective small molecule DOT1L inhibitor, stands as the only DOT1L inhibitor that has been a subject of significant clinical attention. In this context, there is an urgent need to load the armoury of the DOT1L inhibitors with new entries that can maximize the therapeutic benefits of inhibiting this epigenetic target in cancer. HDAC inhibitors as small molecule inhibitors have always been at the forefront of investigations and a number of inhibitors belonging to this class of hydroxamic acids and anilides were tested in diverse malignancies both as single agents as well as the part of combination regimens. While these two classes are expected to continue being evaluated clinically as well as preclinically, it is also anticipated that some initiatives will be taken to probe the candidature of the non-metal chelating class of HDAC inhibitors as a potential therapeutic weapon in cancer. A complete profiling of TMP-269, prototype non-metal chelating HDAC inhibitor, might open an avenue for the construction of similar scaffolds. As the non-metal chelating class is expected to be relatively free of the pharmacological liabilities of the metal chelating class of HDAC inhibitors, safer therapeutics can be established via furnishment of agents that binds weakly with the metals but relies on interactions from other parts of the pharmacophore to afford acceptable levels of isoform inhibition. Progress in the field of LSD1 inhibitors has basically relied on the fragment stitching approach on the chemical architecture of tranylcypromine to access new frameworks capable of producing antiproliferative effects. OPY-1001, IMG-7289, GSK-2879552 and ORY-2001 are the tranylcypromine based LSD1 inhibitors that are undergoing clinical stage investigation in different cancer types. Futuristic attempts are likely to be directed towards the hydrazide type LSD1 inhibitors owing to the striking antitumor potential demonstrated by SP-2509. OTX-015, CPI-0610, BMS-986158, GSK525762, INCB054329, INCB057643, ODM-207, RO-6870810, BAY1238097, CC-90010, AZD-5153, PLX-51107, SF-1126 represents the BET inhibitors in various phases of clinical evaluation. It is quite hopeful that a comprehensive profiling of these BET inhibitors might yield a cancer therapeutic in the longer run. In addition, some preclinical studies have excellently utilized the BET inhibitory scaffolds for installation in the PROTACS model. The optimistic activity profile of the BET inhibitor based degraders can also spur numerous investigations in this direction. To sum up, the armoury of clinical and preclinical epigenetic inhibitors is filled with numerous candidates, however, conclusive benefits can only be ascertained after the completion of all the phases of the clinical trials.

Overall, the findings covered in this perspective present recent advances in the field of epigenetic drug discovery. It is apparent that future research will include parallel programs aimed at the completion of the ongoing clinical studies, initiation of new clinical trials and development of new inhibitors. In addition, numerous brain storming sessions involving expertise of interdisciplinary teams composed of chemist, biologist, and formulation chemist along with researchers well versed with computational aspects of drug design needs to be conducted. Such programs can identify the hurdles and the hindering factors that have halted the clinical growth of several inhibitors of the epigenetic targets and present logical ways to strengthen the candidature of epigenetic targets in context of capacity to generate single target agents.

## Data Availability

Not applicable.

## Abbreviations

PROTACS: Proteolysis targeting chimera
DNMT: DNA methyltransferase
HDAC: Histone deacetylase
LSD: Lysine specific demethylase
EZH2: Enhancer of zeste homolog 2
BET: Bromodomain and extra-terminal motif
DOT1L: Disruptor of telomeric silencing 1-like
CRISPR: Clustered regularly interspaced short palindromic repeats
HMT: Histone methyl transferase
miRNAs: MicroRNAs
TSG: Tumor suppressor genes
HSP: Heat shock protein
ADC: Antibody drug conjugate
FDA: Food and drug administration
CR: Complete response
PR: Partial response
ORR: Overall response rate
OS: Overall survival
CI: Confidence interval
MF: Myelofibrosis
HCC: Hepatocellular carcinoma
CRi: Incomplete blood count recovery
CRp: Incomplete platelets recovery
PRC2: Polycomb repressive complex
RP2D: Recommended phase 2 dose
AE: Adverse events
MTD: Maximum tolerated dose
MLL: Mixed lineage leukemia
R/R: Relapsed/refractory
SD: Stable disease
CTCL: Cutaneous T-Cell lymphoma
DLBCL: Diffuse B-cell lymphoma
HAT: Histone acetyltransferase
DLT: Dose limiting toxicity
NSCLC: Non SCLC
TCL, PTCL: PTCL
CML: Chronic myelogenous leukemia
mCRPC: Metastatic castration-resistant prostate cancer
OR: Objective response
CRPC: Castration-resistant prostate cancer
SCLC: SCLC
NHL: Non-Hodgkin’s lymphoma
HER2−: Human epidermal growth factor receptor 2 negative
HR+: Hormone receptor-positive
TAAEs: Treatment associated adverse events
ADC: Antibody drug conjugates
KDMs: Histone demethylase
SAM: S-adenosyl-methionine
DCR: Disease control rate
CD: Cytidine deaminase
CTC: Circulating tumor cell
MLL-r: MLL rearrangements
HL: Hodgkin’s lymphoma
CBR: Clinical benefit rate
NMPA: National medical product administration
MLFS: Morphological leukemia-free state
NMC: NUT midline carcinoma
TNBC: Triple negative breast cancer
